# Bright IDEAS-YA Skills Training and Psychosocial Outcomes of Young Adults With Cancer: A Randomized Clinical Trial

**DOI:** 10.1001/jamanetworkopen.2026.7997

**Published:** 2026-04-21

**Authors:** Katie A. Devine, Marie Barnett, Kristine A. Donovan, Lora M. A. Thompson, Sharon L. Manne, Julia Kearney, Kristine Levonyan-Radloff, Diana B. Diaz, Isabelle Anderson, Shengguo Li, Pamela Ohman-Strickland, Olle Jane Z. Sahler

**Affiliations:** 1 Pediatric Population Science, Outcomes, and Disparities Research Section, Division of Pediatric Hematology and Oncology, Department of Pediatrics, Rutgers Cancer Institute, New Brunswick, New Jersey; 2 Memorial Sloan Kettering Cancer Center, New York, New York; 3 Moffitt Cancer Center, Tampa, Florida; 4 Mayo Clinic, Rochester Minnesota; 5 Department of Medicine, Rutgers Cancer Institute, New Brunswick, New Jersey; 6 Department of Biometrics, Rutgers Cancer Institute, New Brunswick, New Jersey; 7 Rutgers School of Public Health, Piscataway, New Jersey; 8 University of Rochester Medical Center, Rochester, New York

## Abstract

**Importance:**

Young adults (YAs; ages 18-39 years) diagnosed with cancer face unique psychosocial challenges, including high distress and poor health-related quality of life (HRQOL). There are few efficacious interventions tailored to their needs.

**Objective:**

To evaluate the efficacy of Bright IDEAS-YA, a problem-solving skills training intervention tailored for YAs, in reducing distress and improving HRQOL.

**Design, Setting, and Participants:**

This randomized clinical trial was conducted at 3 academic medical centers and enrolled participants between February 2021 and March 2024. Eligible participants were aged 18 to 39 years, within 4 months of a first cancer diagnosis, and undergoing systemic therapy. Participants completed surveys at baseline and 3, 6, 12, and 24 months.

**Intervention:**

Bright IDEAS-YA is a 6-session, one-on-one intervention that teaches a structured approach to problem-solving. Enhanced usual care (EUC) included standard psychosocial care plus a list of adolescent and YA resources.

**Main Outcomes and Measures:**

Primary outcomes were depression and anxiety (Patient-Reported Outcomes Measurement Information System Short Forms) and HRQOL (Functional Assessment of Cancer Therapy-General) at 6 months. Problem-solving ability (Social Problem-Solving Inventory-Revised Short Form) was examined as a mediator. Analyses used linear mixed effects models and mediation analysis.

**Results:**

Of the 1128 YAs screened, 344 YAs (median [IQR] age, 31.27 [25.78-36.38] years; 251 female [62.5%]) enrolled, with 171 allocated to intervention and 173 allocated to control. Overall, 296 YAs (86.0%) and 280 YAs (81.4%) completed 3- and 6-month surveys, respectively. At 6 months, the intervention group showed significantly greater improvements than EUC in depression (effect size estimate, −3.23 points; 95% CI, −4.93 to −1.53 points; *P* < .001), anxiety (effect size estimate,−2.43 points; 95% CI, −4.05 to −0.81 points; *P* = .003), and HRQOL (effect size estimate, 3.40 points; 95% CI, 0.34 to 6.45 points; *P* = .03). These changes were clinically meaningful. Mediation analyses showed that improvements in depression and anxiety were partially due to increased problem-solving ability, particularly reductions in negative problem orientation. Sex and education were moderators of treatment effect, with the treatment effect on anxiety being greater for males (effect size estimate, −5.29 points; 95% CI, −7.97 to −2.60 points) and the treatment effect on anxiety (effect size estimate, −6.94 points; 95% CI, −10.50 to −3.43 points) and depression (effect size estimate, −7.47 points; 95% CI, −11.20 to −3.74 points; *P* < .001) being greater for those with lower education.

**Conclusions and Relevance:**

In this randomized clinical trial of Bright IDEAS-YA, intervention participants had significantly reduced distress and improved HRQOL relative to control. These findings suggest that Bright IDEAS-YA may be offered as supportive care for YAs with cancer to improve psychosocial outcomes.

**Trial Registration:**

ClinicalTrials.gov Identifier: NCT04585269

## Key Points

**Question:**

Is the Bright IDEAS-Young Adult problem-solving skills training intervention efficacious in reducing symptoms of depression and anxiety and improving health-related quality of life for young adults newly diagnosed with cancer?

**Findings:**

In this randomized clinical trial that included 344 young adults aged 18 to 39 years, those assigned to Bright IDEAS-Young Adult intervention experienced significantly greater improvements in depression, anxiety, and health-related quality of life compared with those assigned to usual psychosocial care and tailored resources.

**Meaning:**

These findings suggest that a brief problem-solving skills training intervention should be considered as part of supportive care to meaningfully improve psychosocial outcomes for young adults newly diagnosed with cancer.

## Teaser

This randomized clinical trial evaluates the efficacy of Bright IDEAS-Young Adults, a problem-solving skills training intervention tailored to young adults with cancer, for reducing distress and improving health-related quality of life.

## Introduction

Young adults (YAs) diagnosed with cancer between the ages of 18 to 39 years face a distinct set of emotional, social, and practical needs.^1^ Young adulthood is a life stage characterized by major developmental milestones, such as establishing independence, pursuing education and careers, forming relationships, and potentially starting families, which can be significantly disrupted by a cancer diagnosis and treatment.^2,3^ Research demonstrates that YA cancer survivors experience unmet physical, emotional, and practical needs both during treatment and in the years that follow.^4,5,6,7^ These unmet needs are associated with heightened emotional distress and diminished health-related quality of life (HRQOL).^2,8^

Despite growing awareness of these needs, there are few evidence-based psychosocial interventions specifically designed for YAs undergoing cancer therapy.^9,10^ The number of psychosocial intervention trials for adolescent and YA cancer survivors has increased in the last decade, with much of the work focusing on feasibility and acceptability.^11^ Evidence from 2 randomized trials of resilience and stress management training demonstrated psychosocial benefits for newly diagnosed adolescent and YA survivors aged 12 to 24 years.^12,13,14^ There is promising feasibility and acceptability evidence for a mindfulness music therapy for YAs receiving cancer treatment,^15^ an eHealth positive psychology skills intervention for posttreatment YA survivors,^16^ as well as a technology-assisted Cognitive Behavioral Therapy for adolescent and YA survivors with moderate depressive symptoms during or posttreatment.^17^ There remains an opportunity for developmentally appropriate interventions that can address a wide range of needs for YAs undergoing cancer therapy and are practical for use during a highly stressful period following a new diagnosis. Problem-solving skills training offers a promising approach because it equips individuals with a flexible, generalizable method for managing a wide range of stressors.^18^ This approach, rooted in problem-solving therapy and cognitive behavioral therapy, has demonstrated effectiveness in improving coping skills, reducing emotional distress, and enhancing HRQOL in both general^18,19,20,21^ and cancer populations.^22,23,24,25,26^

Bright IDEAS is a structured, manualized problem-solving skills training intervention originally developed for caregivers of patients with pediatric cancer.^27^ Delivered one on one by a skilled trainer, the program has shown consistent benefits in multiple randomized trials, including improved problem-solving abilities and reduced symptoms of depression and posttraumatic stress.^27,28,29^ Bright IDEAS teaches a 5-step method (ie, Identify the problem; Define your options; Evaluate options; Act; and See if it worked) in the context of a positive (or bright) problem-solving orientation. The underlying conceptual framework proposes that by improving problem-solving orientation and skills, YAs will be better equipped to identify and act on problems and thus reduce symptoms of distress and improve HRQOL. While the trainer is empathic, they focus on skill teaching and application rather than general psychosocial support.

To better serve YA cancer survivors, we tailored the original Bright IDEAS to this population—Bright IDEAS-YA.^30^ Key modifications included reducing the number of sessions, simplifying materials, incorporating age-relevant examples, and broadening the definition of a problem to include goals and challenges.^30^ A pilot study demonstrated that Bright IDEAS-YA was feasible and acceptable, with strong engagement and satisfaction among participants.^30^ Preliminary outcomes suggested improvements in problem-solving ability and gains in psychosocial outcomes, supporting a fully powered efficacy trial. We designed a randomized trial to evaluate the efficacy of Bright IDEAS-YA compared with enhanced usual care (EUC), consisting of usual psychosocial care plus a list of YA-tailored resources.^31^

Here we report the results of this randomized clinical trial of Bright IDEAS-YA at the primary end point of 6 months postenrollment. The primary aim (aim 1) was to assess the efficacy of Bright IDEAS-YA in reducing symptoms of depression and anxiety and improving HRQOL. The secondary aim (aim 2) was to evaluate whether improvements in problem-solving ability mediated the association of the intervention with outcomes. An exploratory aim (aim 3) was to examine potential moderators of treatment effects, including demographic and baseline levels of financial strain, unmet needs, and distress, which may influence treatment outcomes.

## Methods

### Study Design

This multisite, 2-arm randomized clinical trial evaluated the efficacy of Bright IDEAS-YA compared with enhanced usual psychosocial care. Participants were recruited from 3 academic cancer centers in the US between February 2021 and March 2024, and followed up over 24 months, with assessments at baseline, 3, 6, 12, and 24 months. The study aimed to enroll 344 participants. Single institutional review board approval was obtained from Rutgers Biomedical and Health Sciences institutional review board. The protocol was published with detailed information regarding study design, procedures, measures, and statistical analysis plan (Supplement 1).^31^ Results reporting adheres to the Consolidated Standards of Reporting Trials (CONSORT) reporting guideline.^32^

### Participants and Procedures

Eligible participants were YAs aged 18 to 39 years, within 4 months of a first cancer diagnosis, and undergoing systemic therapy (eg, chemotherapy, radiation, immunotherapy, or stem cell transplant). Exclusion criteria were documented or self-reported cognitive delay or impairment, life expectancy less than 6 months, or treatment limited to surgery. English-language proficiency was required.

Potential participants were identified through electronic health records and clinician referrals. Trained research staff approached eligible individuals in person or remotely, provided study information, and obtained electronic informed consent using a culturally sensitive and participant-centered approach.

Following completion of the baseline survey, participants were randomized in a 1:1 ratio to either the intervention or control group. Randomization was stratified by site and age group (18-29 vs 30-39 years) using variable block sizes designed by the study biostatistician (P.O.S.) and implemented electronically. Participants were alerted of their assignment via email.

### Bright IDEAS-YA Intervention

Bright IDEAS-YA is a 6-session, manualized problem-solving skills training program delivered via secure video sessions by a trainer. Trainers were licensed mental health professionals or trainees who were taught how to deliver the Bright IDEAS program and received ongoing supervision by a licensed psychologist (M.B., K.A. Devine, L.M.A.T., K.A. Donovan). Bright IDEAS is an acronym to help remember the problem-solving approach. Bright communicates a positive orientation toward problems, cultivating a mindset that problems are challenges that can be managed rather than threats. IDEAS is an acronym that refers to the 5 steps in the model: (1) Identify the problem; (2) Define your options; (3) Evaluate options; (4) Act; and (5) See if it worked. Sessions were scheduled weekly but could be extended over 12 weeks to accommodate personal or medical schedules. In session 1, trainers taught the Bright IDEAS model and identified problems or challenges relevant to the participant. In sessions 2 through 5, trainers coached participants to work through their selected problems and challenges using the Bright IDEAS model. The trainer guided the participant to apply the steps to their self-identified challenge using structured worksheets for each step. There was an optional worksheet for challenging distorted thoughts used as appropriate depending on the problem and solutions identified. In session 6, trainers and participants reviewed their progress using Bright IDEAS and discussed strategies to continue using the skills after the program was over. Treatment fidelity was monitored by 3 investigators with expertise in Bright IDEAS and the principal investigator (O.J.S., S.L.M., J.K., and K.A.D.) through review of audio recordings using structured checklists and group discussions.

### EUC Comparison

Participants in the EUC group received standard institutional psychosocial care, which typically consisted of a meeting with a social worker and access to psychosocial support services. They were also mailed a copy of the list of support resources from the National Comprehensive Cancer Network Adolescent and Young Adult Guideline for Patients.^33^

### Measures

Participants completed surveys at baseline and 3, 6, 12, and 24 months. Six months was selected a priori as the primary end point, with other time periods as secondary end points. Only data from baseline, 3 months, and 6 months are presented in this study. Primary outcomes included symptoms of depression and anxiety, assessed via the Patient-Reported Outcomes Measurement Information System (PROMIS) Depression and Anxiety Short Forms,^34^ and HRQOL, assessed via the Functional Assessment of Cancer Therapy-General (FACT-G) version 4.^35^ PROMIS measures yield T-scores, with higher scores indicating higher symptoms of depression and anxiety. The FACT-G yielded an overall score and 4 subscales (physical well-being, social and family well-being, emotional well-being, and functional well-being), with higher scores indicating better HRQOL. The overall HRQOL was used in this analysis.

Change in problem-solving ability at 3 months, measured by the Social Problem-Solving Inventory-Revised Short Form (SPSI-R:SF),^36^ was considered as a mediator for changes in outcomes at 6 months. The SPSI-R:SF yielded an overall score, as well as 5 subscale scores (positive problem orientation, negative problem orientation, rational problem-solving style, impulsive and carelessness style, and avoidant style). Higher total score indicated higher problem-solving ability, while a higher subscale score indicated more of that style. Potential moderators included baseline levels of financial strain, unmet needs, and distress. Financial strain was measured by the Comprehensive Score for Financial Toxicity,^37^ with lower scores indicating greater financial strain. Unmet needs were measured by the Adolescent and Young Adult Oncology Screening Tool,^38^ which was adapted from the National Comprehensive Cancer Network Distress Thermometer and Problem List^39,40^ to include problems relevant to YAs across a variety of domains (eg, practical, emotional, family, and social). A total sum score was calculated.

Participants self-reported race and ethnicity using the US National Institutes of Health categories of American Indian or Alaska Native, Asian, Black or African American, Native Hawaiian or Other Pacific Islander, more than 1 race, other (self-described), or unknown; race and ethnicity were included as required by the funding agency. Participants in the intervention group reported their satisfaction with Bright IDEAS-YA using a 10-item questionnaire derived from the Multi-Dimensional Treatment Satisfaction Measure^41^ assessing utility of intervention-specific components (eg, the user manual and worksheets), attitude toward the intervention, and trainer competence. Items were rated on a 1 to 5 scale, with higher scores indicating greater satisfaction. Perceived benefit attributable to the intervention was rated on a 0 to 100 scale.

### Statistical Analyses

The primary aim, evaluating the efficacy of the Bright IDEAS-YA intervention vs EUC at 6 months, was tested by linear mixed models with maximum likelihood estimation using R version 4.5.0 (R Project for Statistical Computing) with the lme4 package.^42^ All randomized participants, regardless of the extent to which they completed the intervention, were included in intent-to-treat analyses. Site was included as a fixed effect and participant as a random effect. The primary end point was the estimated change from baseline to 6 months in depression, anxiety, and HRQOL, calculated by linear contrasts of regression parameters using the main effect of time and the interaction between time and treatment group from the linear mixed model. Change from baseline to 3 months was examined as a secondary end point. Model assumptions were tested using random-effects plots and residual plots. Missing data were assumed to be missing at random, in which case linear mixed models with maximum likelihood estimation have been shown to yield reliable estimates.^43^ Planned sensitivity analyses conducted included analysis without adjusting for site, per-protocol analysis (including only participants who completed at least 4 of the 6 intervention sessions because this was considered to provide ample experience with the Bright IDEAS model), and adjustment for imbalance in baseline characteristics, if applicable.

For aim 2, mediation was evaluated using the mediation analysis in R.^44^ Change in total SPSI-R:SF score from baseline to postintervention (T1-T2) was analyzed as a potential mediator of treatment effects from baseline to 6 months (T1-T3); then, the subscales were examined. Of note, this analysis differed slightly from the published protocol,^31^ which proposed a latent variable structural model to evaluate problem-solving ability as a latent mediating variable from the 5 subscales. Given that the SPSI-R:SF yields a total score with strong internal reliability (Cronbach α = 0.89), mediation analysis focused on total score was deemed appropriate, with follow-up analyses for the subscales. For aim 3, moderation was evaluated by adding a moderator × group × time interaction variable to the linear mixed models. Potential moderators included biological sex, race, ethnicity, age category (18-29 vs 30-39 years), and baseline levels of financial strain, unmet needs, and distress (dichotomized as high or low using median split). Statistical significance was defined as a 2-sided *P* < .05.

## Results

### Participants

**Figure 1.**
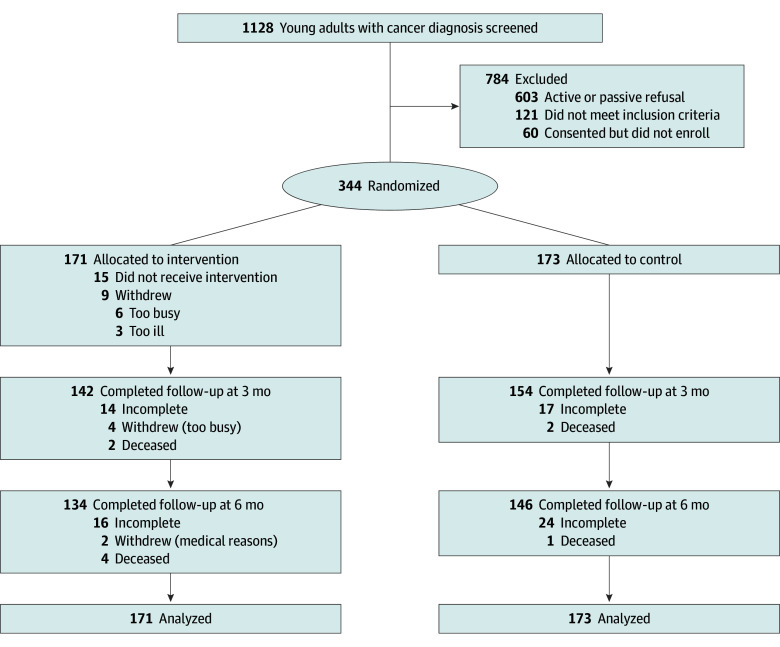
Consolidated Standards of Reporting Trials Flow Diagram

A total of 1128 patients were screened, 121 did not meet eligibility criteria, 603 actively or passively refused, 60 consented but did not enroll, and 344 YAs enrolled (34.2% acceptance rate), with 171 randomized to intervention, and 173 randomized to control. Of the 344 participants, 296 (86.0%) and 280 (81.4%) completed surveys at 3 and 6 months, respectively. After removing those who were deceased or withdrawn for medical reasons, retention rates were 296 of 340 participants (87.1%) at 3 months and 280 of 333 participants (84.1%) at 6 months (Figure 1). Sample demographic and medical characteristics are presented in Table 1. Of all participants, 215 (62.5%) identified as female and 128 (37.2%) identified as male; 43 identified as Asian (12.5%), 38 (11.0%) identified as Black or African American, and 222 (64.5%) identified as White; and the median (IQR) age was 31.27 (25.78-36.38) years. The most common cancer diagnoses included lymphoma, breast cancer, and leukemia. Analysis of demographic information available regarding those who declined participation vs those who enrolled indicated that males were more likely than females to decline participation (χ^2^_1_ = 13.94; *P* < .001) and those with medical record race categorized as Asian, used here because it was the only information available for decliners, were more likely to enroll than all other racial categories (χ^2^_4_ = 10.32; *P* = .04). Once enrolled, there were no statistically significant differences in demographic or baseline levels of outcome measures among those who completed the 3-month surveys vs those who did not complete them. For the 6-month survey, individuals with a high school degree or less were less likely to complete the survey than those with some college or higher education (χ^2^_1_ = 6.75; *P* = .009), and individuals with other cancer diagnoses were less likely to complete the 6-month survey relative to those with blood or breast cancers (χ^2^_2_ = 8.95; *P* = .009) (eAppendix in Supplement 2).

**Table 1.** Participant Characteristics

Characteristic	Participants, No. (%) (N = 344)	
	Control (n = 173)	Intervention (n = 171)
Age, median (IQR), y	31.48 (26.68-36.30)	31.06 (24.62-36.50)
Sex		
Male	68 (39.3)	60 (35.1)
Female	104 (60.1)	111 (64.9)
Other	1 (0.6)	0
Race		
Asian	20 (11.6)	23 (13.5)
Black or African American	17 (9.8)	21 (12.3)
White	117 (67.6)	105 (61.4)
>1 Race	7 (4.1)	6 (3.5)
Other^a^	6 (3.5)	7 (4.1)
Unknown or missing	6 (3.5)	9 (5.3)
Hispanic ethnicity		
Yes	27 (15.6)	37 (21.6)
No	146 (84.4)	134 (78.4)
Employment status		
Student, part time or full time	21 (12.2)	33 (19.4)
Working, part time or full time	135 (78.5)	117 (68.8)
Unemployed	13 (7.6)	15 (8.8)
Homemaker or caregiver	3 (1.7)	5 (2.9)
Missing	1 (0.6)	1 (0.6)
Highest grade completed		
Less than high school, high school, or GED	43 (24.9)	53 (31.2)
2-y College	18 (10.4)	22 (12.9)
4-y College	63 (36.4)	49 (28.8)
Graduate degree	49 (28.3)	46 (27.1)
Missing	0	1 (0.6)
Health insurance		
None	1 (0.6)	4 (2.3)
Public or public and private	26 (15.0)	24 (14.1)
Private	139 (80.4)	138 (80.7)
Military	7 (4.1)	4 (2.3)
Missing	0	1 (0.6)
Income, $		
<50 000	34 (20.4)	28 (17.5)
50 000-99 999	33 (19.8)	40 (25.0)
≥100 000	77 (46.1)	53 (33.1)
Do not know or prefer not to answer	23 (13.8)	39 (24.4)
Missing	6 (3.5)	11 (6.4)
Marital status		
Single, never married	76 (43.9)	79 (46.2)
Married or remarried	65 (37.8)	74 (43.3)
Unmarried but living with partner	24 (13.9)	11 (6.4)
Separated or divorced	8 (4.6)	7 (4.1)
Cancer diagnosis		
Lymphoma (Hodgkin or non-Hodgkin)	45 (26.0)	50 (29.2)
Breast	44 (25.4)	44 (25.7)
Leukemia	17 (9.8)	22 (12.9)
Sarcoma	15 (8.7)	16 (9.4)
Colorectal	16 (9.3)	7 (4.1)
Testicular	9 (5.2)	7 (4.1)
Cervical, ovarian, or endometrial	4 (2.3)	6 (3.5)
Other^b^	23 (13.3)	19 (11.1)
Anxiety T-score at baseline, mean (SD)	58.23 (7.93)	58.93 (7.99)
Depression T-score at baseline, mean (SD)	51.82 (8.90)	53.55 (9.10)
HRQOL at baseline, mean (SD)	71.32 (15.17)	68.96 (16.10)

Abbreviations: GED, general educational development; HRQOL, health-related quality of life.

^a^ Other race included American Indian or Alaska Native, Native Hawaiian or Other Pacific Islander, and self-described write-in responses such as Middle Eastern, Guatemalan, Columbia, Egyptian Coptic, North African, Mexican, Puerto Rican, and Caribbean.

^b^ Other cancer diagnoses include central nervous system tumor, gastric, head and neck, myeloma, neuroendocrine, thoracic or lung, and melanoma or other skin cancers.

### Intervention Completion, Satisfaction, and Fidelity

Of the 171 assigned to the intervention group, 147 (86.0%) completed at least 1 session and 24 (14.0%) did not complete any sessions (mostly due to difficulty scheduling, feeling too ill, or no longer interested). Overall, 123 YAs (71.9%) completed 4 or more sessions and were considered per protocol in sensitivity analyses. There were no statistically significant differences in demographic or baseline levels of outcome measures among those who completed sessions per protocol vs those who did not, except for education. Individuals with a high school degree or less were less likely to complete 4 or more sessions than those with some college or higher education (χ^2^_1_ = 5.52; *P* = .02). Participants reported high satisfaction with the Bright IDEAS-YA intervention across items, with the highest rating for the trainer being warm and supportive (mean [SD] rating, 4.74 [0.63]) and the lowest rating for the usefulness of the worksheets (mean [SD] rating, 4.00 [0.82]). On average, participants reported a mean (SD) 54.57% (26.50%) improvement in coping and daily functioning (median [range], 50.00% [0.00% to 100.00%]) because of the intervention. Treatment fidelity ratings demonstrated a mean (SD) of 93.9% (11.1%) across all sessions rated, demonstrating high trainer fidelity to the intervention manual.

### Efficacy

Table 2 shows that from baseline to 6 months, the intervention group demonstrated significantly greater improvements than EUC in depression (effect size estimate, −3.23 points; 95% CI, −4.93 to −1.53 points; *P* < .001), anxiety (effect size estimate,−2.43 points; 95% CI, −4.05 to −0.81 points; *P* = .003), and HRQOL (effect size estimate, 3.40 points; 95% CI, 0.34 to 6.45 points; *P* = .03). The estimate for depression, for example, indicates that the decrease in the intervention group relative to the control was 3.23 points greater. These changes can be characterized as clinically meaningful based on published reports (ie, 3.0 to 3.1 points for depression, 2.3 to 3.4 points for anxiety, and 3.0 to 7.0 points for HRQOL).^45,46,47^ In the HRQOL model, the control group showed statistically significant improvements from baseline to 6 months only, but the change in the intervention group was more than 2 times greater. The secondary end point of change from baseline to 3 months also showed significant treatment effects in depression (effect size estimate, −2.02 points; 95% CI, −3.69 to −0.36 points; *P* = .02), anxiety (effect size estimate, −1.84 points; 95% CI, −3.43 to −0.25 points; *P* = .02), and HRQOL (effect size estimate, 3.13 points; 0.14 to 6.13 points; *P* = .04).

**Table 2.** Results of Linear Mixed Models Comparing Intervention With Enhanced Usual Care on Primary Outcomes^a^

Analysis	Depression		Anxiety		HRQOL	
	Estimate (95% CI)	*P* value	Estimate (95% CI)	*P* value	Estimate (95% CI)	*P* value
Baseline to 3-mo change						
Control	0.26 (−0.89 to 1.42)	.66	−0.90 (−1.49 to 0.71)	.48	−0.12 (−2.19 to 1.96)	.91
Intervention	−1.76 (−2.96 to −0.56)	.004	−2.23 (−3.38 to −2.09)	<.001	3.02 (0.86 to 5.18)	.006
Treatment effect	−2.02 (−3.69 to −0.36)	.02	−1.84 (−3.43 to −0.25)	.02	3.13 (0.14 to 6.13)	.04
Baseline to 6-mo change						
Control	0.31 (−0.86 to 1.49)	.60	0.22 (−0.90 to 1.34)	.70	2.66 (0.55 to 4.78)	.01
Intervention	−2.92 (−4.14 to −1.69)	<.001	−2.21 (−3.38 to −1.04)	<.001	6.06 (3.85 to 8.27)	<.001
Treatment effect	−3.23 (−4.93 to −1.53)	<.001	−2.43 (−4.05 to −0.81)	.003	3.40 (0.34 to 6.45)	.03

Abbreviation: HRQOL, health-related quality of life.

^a^ Intent-to-treat analyses included all randomized participants (171 intervention and 173 control participants). Depression was measured using the Patient-Reported Outcomes Measurement Information System (PROMIS) Depression T-score, anxiety was measured using the PROMIS Anxiety T-Score, and HRQOL was measured using the Functional Assessment of Cancer Therapy-General Total Score.

### Mediation

**Figure 2.**
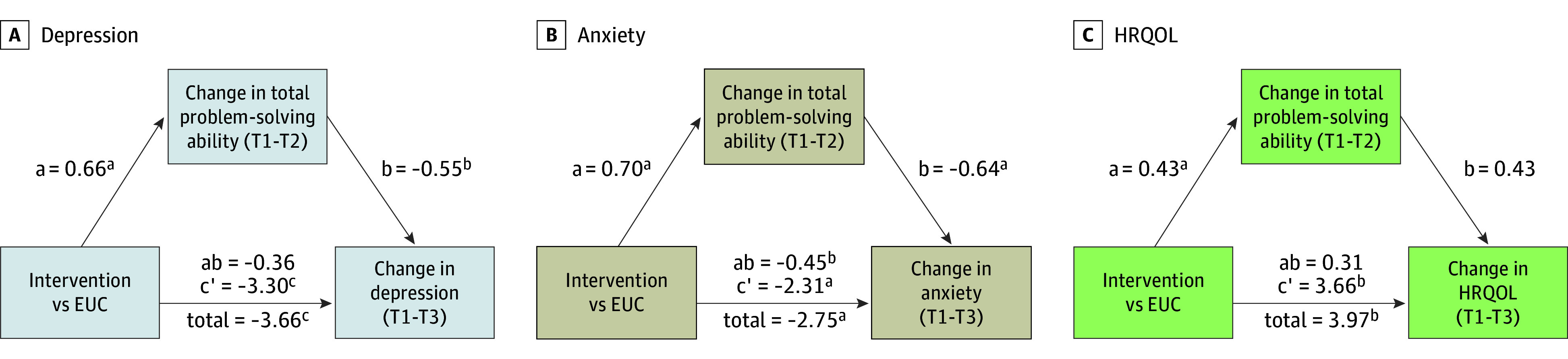
Mediation Analysis Graphs Evaluating Baseline to Postintervention Change in Total Problem-Solving Ability as a Mediator of Treatment Effects on Changes From Baseline to 6 Months (T1-T2) Parameter estimates are unstandardized coefficients. Path a is the direct effect of intervention on the mediator (ie, whether the intervention successfully changed problem-solving ability); path b is the direct effect of the mediator on change in outcome (ie, whether changes in problem-solving ability were associated with changes in the outcome); path ab is the indirect effect of the intervention on change in outcome through the mediator (ie, how much of the outcome change could be explained by the mediator); path c′ is the direct effect of intervention on change in outcome (ie, the effect of the intervention not through the mediator); and total is the total effect (ie, the overall effect of the intervention on the outcome). Total problem-solving ability was measured using the Social Problem-Solving Inventory–Revised Short Form, depression was measured using the Patient-Reported Outcomes Measurement Information System (PROMIS) Depression Short Form, anxiety was measured using the PROMIS Anxiety Short Form, and health-related quality of life (HRQOL) was measured using the Functional Assessment of Cancer Therapy-General. EUC indicates enhanced usual care. ^a^*P* < .01. ^b^*P* < .05. ^c^*P* < .001.

The effect of the intervention on anxiety symptoms at 6 months was partially mediated by change in problem-solving ability from baseline to 3 months. Specifically, the indirect effect of the treatment via problem-solving ability was statistically significant, with a mediated proportion of 16.19% (95% CI, 3.23% to 45.08%). The indirect effect of the intervention on depression was marginally statistically significant, with a mediated proportion of 9.84% (95% CI, 1.21% to 26.49%). There was no statistically significant indirect effect for HRQOL (Figure 2 and eAppendix in Supplement 2). Analyses of the SPSI-R:SF subscales indicated that mediation was primarily due to reductions in negative problem orientation (eAppendix in Supplement 2).

### Moderation

In the models examining sex, race, ethnicity, age category, financial strain, unmet needs, and distress as potential moderators, only the 3-way interaction for sex was marginally statistically significant at 6 months for anxiety (*P* for interaction = .03). The intervention effect was large and significant among males (effect size estimate, −5.29 points; 95% CI, −7.97 to −2.60 points; *P* < .001) but not among females (effect size estimate, −0.75 points; 95% CI, −2.79 to 1.29 points; *P* = .47). In fact, in the control group, males had nonsignificantly increased anxiety (effect size estimate, 1.57 points; 95% CI, −0.21 to 3.35 points; *P* = .08), but males in the intervention group had significantly decreased anxiety (effect size estimate, −3.72 points; 95% CI, −5.73 to −1.71 points; *P* < .001). Females saw a smaller decrease in anxiety under the intervention (effect size estimate, −1.52 points; 95% CI, −2.95 to −0.08 points; *P* = .04) (eAppendix in Supplement 2). None of the other planned 3-way interactions (time × arm × moderator) were statistically significant (eAppendix in Supplement 2).

### Sensitivity Analyses

Analyses without the inclusion of site as a fixed factor were not remarkably different than those including site (eAppendix in Supplement 2). The per-protocol analysis, which excluded participants who did not complete at least 4 of the 6 intervention sessions, demonstrated slightly stronger effect size estimates for depression, anxiety, and HRQOL (eAppendix in Supplement 2). There was a slight imbalance in education between the 2 groups; analyses adjusting for education showed no significant differences in parameter estimates (eAppendix in Supplement 2). Given that 18.6% of the sample did not complete the 6-month survey, with higher attrition among those with lower education, we conducted sensitivity analyses with education as a potential moderator (time × arm × education) and found it was a statistically significant modifier of the treatment effect for depression and anxiety. Stratified analyses by education indicated that participants with lower education showed larger treatment effects on depression (effect size estimate, −7.47 points; 95% CI, −11.20 to −3.74 points; *P* < .001) and anxiety (effect size estimate, −6.94 points; 95% CI, −10.50 to −3.43 points; *P* < .001) than those with higher education (eAppendix in Supplement 2). Therefore, if we had retained more participants with a lower level of education, the overall treatment effect would have been higher than the one observed. We also conducted a sensitivity analysis using extreme case analysis, where all missing data were replaced with the top 25th percentile and lowest 25th percentile as assessing the best and worst outcomes.^48^ We found that the magnitude of results attenuated from 1% to 42% under extreme cases, but were in the same direction as the original model.

## Discussion

The results of this randomized clinical trial provide strong evidence that Bright IDEAS-YA problem-solving skills training intervention is efficacious in improving psychosocial outcomes among YAs newly diagnosed with cancer. At the 6-month follow-up, participants in the intervention group demonstrated statistically significant and clinically meaningful reductions in symptoms of depression and anxiety, and improvements in HRQOL, compared with those receiving usual care and YA-tailored resources.

The observed treatment effects of 3.23 points in depression and 2.43 points in anxiety align with or exceed thresholds considered clinically meaningful in published literature.^45,46,49^ Similarly, the 3.40-point difference in FACT-G total score aligns with published literature demonstrating minimally important differences in HRQOL.^36^ It is encouraging to see clinically meaningful change with a relatively brief, 6-session intervention. The long-term end points at 12 and 24 months will assess the durability of these improvements.

Importantly, mediation analyses revealed that improvements in problem-solving ability accounted for a statistically significant proportion of the observed treatment effect on anxiety, with reductions in the negative problem orientation subscale emerging as the underlying key factor. This finding is consistent with prior trials of Bright IDEAS with caregivers of pediatric patients, which also found the reduction in negative problem orientation was a key mechanism of outcomes, as was a reduction in avoidant strategies.^28^ These partial mediation results also support the theoretical foundations of Bright IDEAS-YA, which focus on enhancing positive problem-orientation and rational problem-solving skills, while reducing negative problem-orientation and irrational or avoidant strategies, to enhance overall coping and improve psychosocial outcomes. The repeated emphasis on reframing problems, generating solutions, evaluating outcomes, and reinforcing success over the 6 sessions appears to be important in helping YAs manage the complex stressors associated with cancer diagnosis and treatment and reducing negative affect; this aligns well with results of a recent trial of resilience and stress management training for adolescent and YAs with advanced cancer, which indicated that building coping and resilience strategies were important for future improvements in anxiety, depression, and HRQOL.^13^

Treatment effects on anxiety at 6 months were found to be greater for males. In contrast to the literature generally showing female sex to be associated with increased distress,^50^ in this trial, males in the control group demonstrated increased anxiety over time, but decreased anxiety under the intervention. This finding calls for further work to facilitate psychosocial intervention uptake among males, who enrolled at lower rates than females in this trial and other psychosocial research.^51^ The lack of other baseline factors emerging as moderators suggests broad utility of Bright IDEAS-YA. Thus, Bright IDEAS-YA may be offered to all YAs, regardless of background characteristics or presenting levels of financial strain, unmet needs, or distress; this, again, aligns with the underlying framework of Bright IDEAS in that supporting problem-solving can enhance coping and reduce distress, regardless of the types of problems or challenges encountered.

Sensitivity analyses demonstrated that those who completed at least 4 of the 6 intervention sessions demonstrated greater benefit than those who completed less than 4 sessions. While over 70% of our intervention sample completed at least 4 sessions, a subgroup was unable, predominantly due to feeling unwell and scheduling difficulties. Although we offered flexibility in scheduling, rescheduling, and telehealth visits, this highlights the complexity of working with patients in the first few months postdiagnosis. Findings demonstrated that individuals with lower educational attainment had reduced completion rates for both the intervention and the 6-month assessment, highlighting potential barriers to participation in this group. Despite these challenges, the intervention produced larger effects among participants with lower educational backgrounds. Future efforts should focus on developing tailored strategies to support sustained engagement among YAs with lower educational attainment.

These results have several implications for clinical practice and future research. First, Bright IDEAS-YA offers a brief evidence-based intervention that can be integrated into routine cancer care for YAs. Its manualized approach can be learned by any psychosocial staff and does not require licensure to deliver. Its structured and skills-based approach may be appealing to individuals hesitant to engage in traditional mental health services, which have been underutilized by YAs.^52,53^ Second, the delivery of Bright IDEAS-YA via videoconference was well received by YAs, as evidenced by high attendance and satisfaction ratings. This telehealth approach may enhance the scalability of the intervention. Third, the identification of negative problem orientation as a key mechanism of action on negative affect outcomes suggests that future refinements of the intervention could place even greater emphasis on reframing problems as challenges that can be overcome rather than as overwhelming threats.

### Limitations

Limitations include enrollment of only English-speaking YAs and patients from academic cancer centers, which is not representative of YAs seen in community oncology settings. Future work is needed to identify implementation strategies to bring this intervention to community settings and enhance engagement with YAs with lower educational attainment. While training to be a Bright IDEAS-YA interventionist does not require professional licensure, it does require time and effort to deliver, which may be difficult in busy clinical settings. Just over 70% of our sample were able to complete the intervention, with those unable being too ill or busy; this highlights the need for a flexible, individualized approach to delivery. Our acceptance rate of 34% reflects challenges enrolling YA cancer survivors in multisite psychosocial intervention trial research. A recent systematic review found wide variability in enrollment rates among studies recruiting YA cancer survivors ages 15 to 39 years, from 4.7% to 79%,^54^ and our study aligned with other recent behavioral intervention trials.^13,16,17^ We found that males were less likely to enroll than females, which is consistent with other work,^51^ but highlights a need to better tailor psychosocial interventions to men. The heterogeneity of cancer diagnoses may be seen as both a limitation and a strength; the small subgroups prohibit examination of any differences by disease group but the inclusion of the spectrum of diagnoses affecting YAs enhances the generalizability of our findings.

## Conclusions

In this randomized clinical trial of Bright IDEAS-YA, intervention participants experienced significantly reduced symptoms of depression and anxiety and improved HRQOL relative to control participants. Reduction in anxiety symptoms were partially attributed to improvement in problem-solving ability. These findings support the broader adoption of structured, skills-based psychosocial interventions to address the unmet needs of this vulnerable population. Future work is needed to identify barriers and facilitators to implementing this intervention in other settings, particularly in community-based oncology practices where most YAs might be treated for cancer.

## Notes

Supplement 1. Trial Protocol and Statistical Analysis Plan

Supplement 2. eAppendix. Supplementary Data

Supplement 3. Data Sharing Statement

## References

[1] Bleyer WA. Cancer in older adolescents and young adults: epidemiology, diagnosis, treatment, survival, and importance of clinical trials. Med Pediatr Oncol. 2002;38(1):1-10. doi:10.1002/mpo.1257 11835231

[2] Devins GM, Bezjak A, Mah K, Loblaw DA, Gotowiec AP. Context moderates illness-induced lifestyle disruptions across life domains: a test of the illness intrusiveness theoretical framework in six common cancers. Psychooncology. 2006;15(3):221-233. doi:10.1002/pon.940 15996006

[3] Smith AW, Bellizzi KM, Keegan THM, . Health-related quality of life of adolescent and young adult patients with cancer in the United States: the adolescent and young adult health outcomes and patient experience study. J Clin Oncol. 2013;31(17):2136-2145. doi:10.1200/JCO.2012.47.3173 23650427 PMC3731979

[4] Jones JM, Fitch M, Bongard J, . The needs and experiences of post-treatment adolescent and young adult cancer survivors. J Clin Med. 2020;9(5):1444. doi:10.3390/jcm9051444 32413981 PMC7291222

[5] Smith AW, Parsons HM, Kent EE, ; AYA HOPE Study Collaborative Group. Unmet support service needs and health-related quality of life among adolescents and young adults with cancer: the AYA HOPE study. Front Oncol. 2013;3:75. doi:10.3389/fonc.2013.00075 23580328 PMC3619248

[6] Smith AW, Keegan T, Hamilton A, ; AYA HOPE Study Collaborative Group. Understanding care and outcomes in adolescents and young adult with Cancer: a review of the AYA HOPE study. Pediatr Blood Cancer. 2019;66(1):e27486. doi:10.1002/pbc.27486 30294882 PMC7239374

[7] Zebrack B, Isaacson S. Psychosocial care of adolescent and young adult patients with cancer and survivors. J Clin Oncol. 2012;30(11):1221-1226. doi:10.1200/JCO.2011.39.5467 22412147

[8] Zebrack BJ. Psychological, social, and behavioral issues for young adults with cancer. Cancer. 2011;117(10)(suppl):2289-2294. doi:10.1002/cncr.26056 21523748

[9] McGrady ME, Willard VW, Williams AM, Brinkman TM. Psychological outcomes in adolescent and young adult cancer survivors. J Clin Oncol. 2024;42(6):707-716. doi:10.1200/JCO.23.01465 37967297 PMC13019686

[10] Thornton CP, Ruble K, Kozachik S. Psychosocial interventions for adolescents and young adults with cancer: an integrative review. J Pediatr Oncol Nurs. 2020;37(6):408-422. doi:10.1177/1043454220919713 32452711

[11] Murphy KM, Siembida E, Lau N, Berkman A, Roth M, Salsman JM. A systematic review of health-related quality of life outcomes in psychosocial intervention trials for adolescent and young adult cancer survivors. Crit Rev Oncol Hematol. 2023;188:104045. doi:10.1016/j.critrevonc.2023.104045 37269881 PMC10527433

[12] Rosenberg AR, Bradford MC, McCauley E, . Promoting resilience in adolescents and young adults with cancer: results from the PRISM randomized controlled trial. Cancer. 2018;124(19):3909-3917. doi:10.1002/cncr.31666 30230531

[13] Rosenberg AR, Fladeboe KM, Zhou C, . Promoting resilience in stress management: a randomized controlled trial of a novel psychosocial intervention for adolescents and young adults with advanced cancer. JCO Oncol Pract. 2026;22(2):243-254. doi:10.1200/OP-25-00161 40294356 PMC12788802

[14] Rosenberg AR, Zhou C, Bradford MC, . Assessment of the promoting resilience in stress management intervention for adolescent and young adult survivors of cancer at 2 years: secondary analysis of a randomized clinical trial. JAMA Netw Open. 2021;4(11):e2136039. doi:10.1001/jamanetworkopen.2021.36039 34817581 PMC8613597

[15] Knoerl R, Mazzola E, Woods H, . Exploring the feasibility of a mindfulness-music therapy intervention to improve anxiety and stress in adolescents and young adults with cancer. J Pain Symptom Manage. 2022;63(4):e357-e363. doi:10.1016/j.jpainsymman.2021.11.013 34896280

[16] Salsman JM, McLouth LE, Tooze JA, . An eHealth, positive emotion skills intervention for enhancing psychological well-being in young adult cancer survivors: results from a multi-site, pilot feasibility trial. Int J Behav Med. 2023;30(5):639-650. doi:10.1007/s12529-023-10162-5 36890329 PMC10485177

[17] Zhang A, Weaver A, Walling E, . Evaluating an engaging and coach-assisted online cognitive behavioral therapy for depression among adolescent and young adult cancer survivors: a pilot feasibility trial. J Psychosoc Oncol. 2023;41(1):20-42. doi:10.1080/07347332.2021.2011530 35040368 PMC10599691

[18] D’Zurilla TJ, Nezu AM. Problem-solving therapy: A positive approach to clinical intervention. 3rd ed. Springer Publishing Co; 2007.

[19] Bell AC, D’Zurilla TJ. Problem-solving therapy for depression: a meta-analysis. Clin Psychol Rev. 2009;29(4):348-353. doi:10.1016/j.cpr.2009.02.003 19299058

[20] Malouff JM, Thorsteinsson EB, Schutte NS. The efficacy of problem solving therapy in reducing mental and physical health problems: a meta-analysis. Clin Psychol Rev. 2007;27(1):46-57. doi:10.1016/j.cpr.2005.12.005 16480801

[21] Cuijpers P, de Wit L, Kleiboer A, Karyotaki E, Ebert DD. Problem-solving therapy for adult depression: an updated meta-analysis. Eur Psychiatry. 2018;48(1):27-37. doi:10.1016/j.eurpsy.2017.11.006 29331596

[22] Syrjala KL, Yi JC, Artherholt SB, . An online randomized controlled trial, with or without problem-solving treatment, for long-term cancer survivors after hematopoietic cell transplantation. J Cancer Surviv. 2018;12(4):560-570. doi:10.1007/s11764-018-0693-9 29730827 PMC6054554

[23] Lee YH, Chiou PY, Chang PH, Hayter M. A systematic review of the effectiveness of problem-solving approaches towards symptom management in cancer care. J Clin Nurs. 2011;20(1-2):73-85. doi:10.1111/j.1365-2702.2010.03401.x 21044188

[24] Blanco C, Markowitz JC, Hellerstein DJ, . A randomized trial of interpersonal psychotherapy, problem solving therapy, and supportive therapy for major depressive disorder in women with breast cancer. Breast Cancer Res Treat. 2019;173(2):353-364. doi:10.1007/s10549-018-4994-5 30343455 PMC6391220

[25] Nezu AM, Nezu CM, Felgoise SH, McClure KS, Houts PS. Project genesis: assessing the efficacy of problem-solving therapy for distressed adult cancer patients. J Consult Clin Psychol. 2003;71(6):1036-1048. doi:10.1037/0022-006X.71.6.1036 14622079

[26] Balck F, Zschieschang A, Zimmermann A, Ordemann R. A randomized controlled trial of problem-solving training (PST) for hematopoietic stem cell transplant (HSCT) patients: effects on anxiety, depression, distress, coping and pain. J Psychosoc Oncol. 2019;37(5):541-556. doi:10.1080/07347332.2019.1624673 31304890

[27] Sahler OJ, Varni JW, Fairclough DL, . Problem-solving skills training for mothers of children with newly diagnosed cancer: a randomized trial. J Dev Behav Pediatr. 2002;23(2):77-86. doi:10.1097/00004703-200204000-00003 11943969

[28] Sahler OJ, Fairclough DL, Phipps S, . Using problem-solving skills training to reduce negative affectivity in mothers of children with newly diagnosed cancer: report of a multisite randomized trial. J Consult Clin Psychol. 2005;73(2):272-283. doi:10.1037/0022-006X.73.2.272 15796635

[29] Sahler OJ, Dolgin MJ, Phipps S, . Specificity of problem-solving skills training in mothers of children newly diagnosed with cancer: results of a multisite randomized clinical trial. J Clin Oncol. 2013;31(10):1329-1335. doi:10.1200/JCO.2011.39.1870 23358975 PMC3607672

[30] Viola AS, Kwok G, Levonyan-Radloff K, . Feasibility and acceptability of Bright IDEAS-Young Adults: a problem-solving skills training intervention. Cancers (Basel). 2022;14(13):3124. doi:10.3390/cancers14133124 35804896 PMC9264826

[31] Devine KA, Ohman-Strickland P, Barnett M, . Protocol of a multisite randomized controlled trial of Bright IDEAS-Young Adults: problem-solving skills training to reduce distress among young adults with Cancer. Contemp Clin Trials. 2024;145:107656. doi:10.1016/j.cct.2024.107656 39111386 PMC11848848

[32] Hopewell S, Chan A-W, Collins GS, . CONSORT 2025 explanation and elaboration: updated guideline for reporting randomised trials. BMJ. 2025;389:e081124. doi:10.1136/bmj-2024-081124 40228832 PMC11995452

[33] National Comprehensive Cancer Network. NCCN guidelines: adolescent and young adult (AYA) oncology. Accessed March 16, 2026. https://www.nccn.org/guidelines/guidelines-detail?category=4&id=1412 10.6004/jnccn.2023.0040 37549914

[34] Pilkonis PA, Choi SW, Reise SP, Stover AM, Riley WT, Cella D; PROMIS Cooperative Group. Item banks for measuring emotional distress from the Patient-Reported Outcomes Measurement Information System (PROMIS®): depression, anxiety, and anger. Assessment. 2011;18(3):263-283. doi:10.1177/1073191111411667 21697139 PMC3153635

[35] Brucker PS, Yost K, Cashy J, Webster K, Cella D. General population and cancer patient norms for the Functional Assessment of Cancer Therapy-General (FACT-G). Eval Health Professions. 2005;28(2):192-211. doi:10.1177/0163278705275341 15851773

[36] D’Zurilla T, Nezu A, Maydeu-Olivares A. Social problem-solving inventory-revised (SPSI-R): Technical manual. Multi-Health Systems; 2002.

[37] de Souza JA, Yap BJ, Hlubocky FJ, . The development of a financial toxicity patient-reported outcome in cancer: the COST measure. Cancer. 2014;120(20):3245-3253. doi:10.1002/cncr.28814 24954526

[38] Patterson P, D’Agostino NM, McDonald FEJ, ; International AYA Cancer Distress Screening Group. Screening for distress and needs: findings from a multinational validation of the Adolescent and Young Adult Psycho-Oncology Screening Tool with newly diagnosed patients. Psychooncology. 2021;30(11):1849-1858. doi:10.1002/pon.5757 34160847 PMC9291177

[39] Holland JC, Bultz BD; National comprehensive Cancer Network (NCCN). The NCCN guideline for distress management: a case for making distress the sixth vital sign. *J Natl Compr Canc Netw*. 2007;5(1):3-7 17323529

[40] National Comprehensive Cancer Network. NCCN Guidelines: Distress Management. Accessed March 16, 2026. https://www.nccn.org/guidelines/guidelines-detail?category=3&id=1431

[41] Sidani S, Epstein DR, Fox M. Psychometric evaluation of a multi-dimensional measure of satisfaction with behavioral interventions. Res Nurs Health. 2017;40(5):459-469. doi:10.1002/nur.21808 28857205 PMC5657530

[42] Bates D, Mächler M, Bolker B, Walker S. Fitting linear mixed-effects models using lme4. J Stat Softw. 2015;67:1-48. doi:10.18637/jss.v067.i01

[43] Chakraborty H, Gu H. A Mixed Model Approach for Intent-to-Treat Analysis in Longitudinal Clinical Trials With Missing Values. RTI Press; 2009. 30896910

[44] Tingley D, Yamamoto T, Hirose K, Keele L, Imai K. Mediation: R package for causal mediation analysis. J Stat Softw. 2014;59:1-38. doi:10.18637/jss.v059.i0526917999

[45] Kroenke K, Stump TE, Chen CX, . Responsiveness of PROMIS and Patient Health Questionnaire (PHQ) Depression Scales in three clinical trials. Health Qual Life Outcomes. 2021;19(1):41. doi:10.1186/s12955-021-01674-3 33541362 PMC7860196

[46] Lee AC, Driban JB, Price LL, Harvey WF, Rodday AM, Wang C. Responsiveness and minimally important differences for 4 patient-reported outcomes measurement information system short forms: physical function, pain interference, depression, and anxiety in knee osteoarthritis. J Pain. 2017;18(9):1096-1110. doi:10.1016/j.jpain.2017.05.001 28501708 PMC5581239

[47] Webster KA, Peipert JD, Lent LF, Bredle J, Cella D. The functional assessment of chronic illness therapy (FACIT) measurement system: guidance for use in research and clinical practice. In: Kassianos AP, ed. Handbook of Quality of Life in Cancer. Springer International Publishing; 2022:79-104.

[48] Council NR. The Prevention and Treatment of Missing Data in Clinical Trials. The National Academies Press; 2010:162. 24983040

[49] Cook KF, Jensen SE, Schalet BD, . PROMIS measures of pain, fatigue, negative affect, physical function, and social function demonstrated clinical validity across a range of chronic conditions. J Clin Epidemiol. 2016;73:89-102. doi:10.1016/j.jclinepi.2015.08.038 26952842 PMC5131708

[50] Osmani V, Hörner L, Klug SJ, Tanaka LF. Prevalence and risk of psychological distress, anxiety and depression in adolescent and young adult (AYA) cancer survivors: a systematic review and meta-analysis. Cancer Med. 2023;12(17):18354-18367. doi:10.1002/cam4.6435 37559504 PMC10523984

[51] Vlooswijk C, Poll-Franse LVV, Janssen SHM, . Recruiting adolescent and young adult cancer survivors for patient-reported outcome research: experiences and sample characteristics of the SURVAYA study. Curr Oncol. 2022;29(8):5407-5425. doi:10.3390/curroncol29080428 36005166 PMC9406992

[52] Zebrack BJ, Corbett V, Embry L, . Psychological distress and unsatisfied need for psychosocial support in adolescent and young adult cancer patients during the first year following diagnosis. Psychooncology. 2014;23(11):1267-1275. doi:10.1002/pon.3533 24664958

[53] Kent EE, Smith AW, Keegan TH, . Talking about cancer and meeting peer survivors: social information needs of adolescents and young adults diagnosed with cancer. J Adolesc Young Adult Oncol. 2013;2(2):44-52. doi:10.1089/jayao.2012.0029 23781400 PMC3684139

[54] Wang RR, Schweitzer JB, Hernandez S, Molina SC, Keegan THM. Strategies for recruitment and retention of adolescent and young adult cancer patients in research studies. J Clin Trans Sci. Published online November 7, 2023. doi:10.1017/cts.2023.669 PMC10663769 38028342

