# Supplementary material for: Bright IDEAS-YA Skills Training and Psychosocial Outcomes of Young Adults With Cancer: A Randomized Clinical Trial
Source: JAMA Netw Open. 2026 Apr 21;9(4):e267997. doi: 10.1001/jamanetworkopen.2026.7997 (PMC13100872; doi:10.1001/jamanetworkopen.2026.7997)
Supplement: Supplement 1. — Trial Protocol and Statistical Analysis Plan [file jamanetwopen-e267997-s001.pdf]

RUTGERS
THE STATE UNIVERSITY OF NEW JERSEY

INTERVENTIONAL RESEARCH PROTOCOL TEMPLATE

(HRP-503a)

STUDY INFORMATION

- **Title of Project:**
  Bright IDEAS-Young Adults: Problem-Solving Skills Training to Reduce Distress among Young Adults with Cancer

- **Principal Investigator Name**
  Katie Devine, PhD, MPH

- **Principal Investigator Div. & Dept.**
  Division of Pediatric Hematology/Oncology, Department of Pediatrics

- **Principal Investigator Contact Info:**
  Katie.devine@rutgers.edu
  195 Little Albany Street
  732-235-7549

- **Protocol Version and Date:**
  V10 2025-2-18

RUTGERS
THE STATE UNIVERSITY OF NEW JERSEY

Table of Contents

Skip To Section: Hold **CTRL** + **Click (Below)** To Follow Link in **Blue**

**1.0**	Research Design
1.1	Purpose/Specific Aims
1.2	Research Significance
1.3	Research Design and Methods
1.4	Preliminary Data
1.5	Sample Size Justification
1.6	Study Variables
1.7	Drugs/Devices/Biologics
1.8	Primary Specimen Collection
1.9	Interviews, Focus Groups, or Surveys
1.10	Timetable/Schedule of Events
**2.0**	Project Management
2.1	Research Staff and Qualifications
2.2	Resources Available
2.3	Research Sites
**3.0**	Multi-Site Research Communication & Coordination
3.1	Outside Research
**4.0**	Research Data Source/s
4.1	Primary Data – Subjects and Specimens
4.2	Subject Selection and Enrollment Considerations
4.3	Subject Randomization
4.4	Secondary Subjects
4.5	Number of Subjects
4.6	Consent Procedures
4.7	Special Consent Populations
4.8	Economic Burden and/or Compensation For Subjects
4.9	Risks to Subjects
4.10	Secondary Data – Record/Chart Reviews, Databases, Tissue Banks, Etc.
4.11	Chart/Record Review Selection
4.12	Secondary Specimen Collection
**5.0**	Special Considerations
5.1	Health Insurance Portability and Accountability Act (HIPAA)
5.2	Family Educational Rights and Privacy Act (FERPA)
5.3	Code of Federal Regulations Title 45 Part 46 (Vulnerable Populations)
5.4	General Data Protection Regulation (GDPR)
5.5	NJ Access to Medical Research Act
**6.0**	Research Data Protection and Reporting
6.1	Data Management and Confidentiality
6.2	Data Security
6.3	Data Safety And Monitoring
6.4	Reporting Results
6.5	Data Sharing
**7.0**	Data and/or Specimen Banking
**8.0**	Other Approvals/Authorizations
**9.0**	Bibliography

1.0 Research Design

1.1 Purpose/Specific Aims

The purpose of this project is to evaluate efficacy of Bright IDEAS, an evidence-based problem-solving skills training (PSST) program, as a supportive care intervention for adolescent and young adult (AYA) cancer patients compared with enhanced usual psychosocial care with 344 young adult patients newly diagnosed with cancer.

Supplemental work will:

- examine the extent to which survivorship care for young adults (ages 18-39) with cancer align with the recently developed National Standards for Survivorship Care and identify barriers and facilitators to obtaining high- quality survivorship care in this group,
- identify barriers and facilitators of disseminating Bright IDEAS-YA in community practices and address barriers to access by culturally and linguistically translating Bright IDEAS-YA for Hispanic, Spanish-speaking young adults.

A. Objectives

We will evaluate the efficacy of Bright IDEAS-YA by examining changes in psychosocial outcomes from baseline to post-intervention (3 months) and follow-up (6, 12, and 24 months).

We will determine the extent to which changes in aspects of problem-solving ability mediate the intervention effects and examine relevant moderators.

B. Hypotheses / Research Question(s)

Aim 1: Evaluate the efficacy of Bright IDEAS-Young Adults on psychosocial outcomes.

Hypothesis 1: Participants who receive the Bright IDEAS-YA intervention will report lower depression, anxiety, and impairment in health-related quality of life over time than participants who receive usual psychosocial care.

Aim 2: Determine the extent to which problem-solving ability mediates treatment effects.

Hypothesis 2: The effects of the intervention will be mediated by improved positive problem orientation and rational problem-solving skills, and reduced negative problem orientation, impulsive style, and avoidant style.

Aim 3 (Exploratory): Examine moderators of treatment effects.

Sex, financial strain, and baseline unmet needs will be examined as potential moderators of treatment effects to identify subgroups who benefit most from this intervention.

Aim 4. (Administrative Supplement) Determine the extent to which survivorship care for young adults aligns with the National Standards for Cancer Survivorship Care. Quantitative items assessing current care experiences will be supplemented with in-depth individual interviews (n = 30) assessing patient perceptions of existing health-care processes aligned with the standards. We expect variation in the perceived quality of care across standards. Results will be used to guide future intervention work.

Aim 5: (Extension Supplement) Identify barriers and facilitators to implementing Bright IDEAS-YA in community practices and address barriers to access by culturally and linguistically translating Bright IDEAS-YA for Hispanic, Spanish-speaking young adults.

1.2 Research Significance

Young adults with cancer diagnosed between the ages of 18 to 39 are increasingly recognized as a vulnerable group with unique emotional, social, and practical needs due to the intersection of cancer treatment and normal developmental processes.[1] Although a cancer diagnosis at any age is highly stressful, cancer diagnosis and treatment during the critical period of young adulthood is particularly challenging. A cancer diagnosis during this time can hinder the process of achieving desired developmental tasks in all life domains, including identity, education, career, financial independence, relationships, and starting a family.[2,3] These disruptions put young adults at particularly high risk of negative outcomes due to their underdeveloped problem-solving ability (i.e., capacity to find effective

solutions to problems) and limited resources (i.e., less experience dealing with similar challenges, unstable social support). Ongoing concerns and unmet informational, emotional, and practical support needs are associated with increased emotional distress and poorer health-related quality of life.[2,4] A critical gap in the field is the lack of evidence-based interventions to address the unique concerns of the young adult population.

To address this clinical care gap, a behavioral intervention is needed that provides skills to manage the diverse and numerous stressors associated with a cancer diagnosis in the context of life transitions, addresses underdeveloped problem-solving ability characteristic of this age group, and is relatively simple to learn and use during the highly stressful time following cancer diagnosis. Problem-solving skills training is uniquely suited to fit this need, as it teaches a global life skill to help young adults address *any* concern, is tailored to individual needs, and is relatively simple to learn and use. Problem-solving skills training is an application of problem-solving therapy, which has accumulated a large body of evidence showing it successfully improves problem-solving ability, reduces negative affect, and improves health-related quality of life.[5]

Young adult survivors are at risk for cancer- and treatment-related late and long-term physical, emotional, and psychosocial effects that require ongoing surveillance, monitoring, and treatment.5 Lifelong survivorship care to address these needs is critical, yet there are many variations in how this care is delivered, including oncology-led, primary care-led, and shared care.[6] Particularly in the United States, where the healthcare system is fragmented and challenging to navigate, it is important to focus on the quality of survivorship care being delivered. To address that gap, the National Cancer Institute and the Department of Veterans Affairs recently created the National Standards for Cancer Survivorship Care that can be used by health systems to develop or enhance survivorship services to meet the needs of cancer survivors.[7] These standards can be adapted to meet the needs of specific populations, such as young adults. In the context of the ongoing parent award, there is an opportunity to identify how well current survivorship services at three comprehensive cancer centers align with the National Standards for Cancer Survivorship Care as applied to the unique needs of young adults transitioning into survivorship care. Emerging literature suggests that the majority of young adult cancer survivors report unmet physical, emotional, and practical needs in the first few years post-treatment.[8-11] Qualitative work in the United States also demonstrates significant ongoing psychosocial needs of young adult cancer survivors and disruption across multiple domains of functioning.[12-15] This work is limited by sampling issues (e.g., including only <25 years) and by focusing on the needs of survivors without explicitly identifying barriers and facilitators to obtaining high quality survivorship care.[12-15] Therefore, there remains a critical gap in understanding how current survivorship care practices align with the new National Standards for Cancer Survivorship Care and young adults' perceptions of barriers and facilitators to obtaining the high-quality care outlined by these standards. We will gather rich qualitative data from a subset (n = 30) of young adults enrolled on the parent award at the time of their transition into survivorship care, rather than retrospectively recalling the transition.

The parent award will determine the efficacy of bright IDEAS-YA as delivered in English at three academic health centers. However, most young adults are treated in community settings.[16] There is often a gap in translating efficacious behavioral interventions into routing clinical care. Therefore, the parent award will be extended to identify strategies to enhance the reach of the intervention and identify barriers and facilitators to implementation in other settings. The RE-AIM framework will guide the approach to consider key factors to optimize the impact of Bright IDEAS-YA for young adults in various settings. We will also culturally and linguistically tailor Bright IDEAS-YA for Hispanic/Spanish-speaking young adults to enhance reach. In the past decade, the largest growing group in the U.S. are those who identify as Hispanic.[17] The majority of adults in the U.S. who are Hispanic speak Spanish (75% can carry a conversation at least pretty well), though those born in the U.S. who are 3rd generation or higher are less likely.[13] Thus, linguistic adaptation into Spanish is important for a subset of young adults, particularly foreign-born or those with fewer generations in the U.S. Additionally, there may be a greater need among this group, as prior research shows that young adult Hispanic cancer survivors report

higher levels of distress at diagnosis than non-Hispanic survivors.[8] Further, young adults who are Hispanic are less likely to receive care at specialized cancer centers, emphasizing the importance of cultural adaptation to enhance effectiveness in real-world settings. Together, these results will yield essential information to move from efficacy to effectiveness.

1.3 Research Design and Methods

Bright IDEAS-YA will be tested with young adult cancer patients in a two-arm parallel randomized controlled trial (RCT) of the intervention versus enhanced usual psychosocial care.

A. Research Procedures

Research staff will collect participants' contact information for tracking and study management. Following consent, baseline surveys (see section 1.9 Data Collection) will be administered online using DatStat, a HIPAA-compliant electronic data capture system. Research staff will either provide a tablet for patients to complete the online survey (paper copies will be available as back-up in the event of significant internet disruption) or email/text a link for completion. If a participant does not complete the baseline survey during an initial in-person visit, we will email a link for completion. After completion of the baseline survey, participants will be randomly assigned 1:1 to either the intervention or enhanced usual psychosocial care comparison group. The study biostatistician will determine a randomization scheme stratified by site, age group (18-29 vs. 30-39 based on definitions of emerging adults and young adults[18])using an undisclosed block size to balance group assignment within each site and age group. This schema will be uploaded to the DatStat program such that completion of the baseline survey will trigger assignment according to the schema via a message to study staff. Participants will then be notified of assignment via an automatic Welcome email through DatStat. For those assigned to the intervention group, study staff will assign a Bright IDEAS-YA trainer, schedule for the participant and trainer to meet at the patient's next medical visit or schedule a virtual meeting to complete the first Bright IDEAS-YA session, and mail/email Bright IDEAS-YA manual, worksheets and the list of resources from the NCCN adolescent and young adult patient guildeline.[19] For those assigned to the enhanced usual psychosocial care group, study staff will mail/email the list of resources from the NCCN adolescent and young adult patient guidelines.[19] All participants will also be mailed a small gift (e.g., Bright IDEAS stress ball) as a token of appreciation for their time and effort. Trainers will schedule all subsequent sessions directly with participants. All participants will be asked to complete surveys at 3 months (post-intervention), 6 months (3-month follow-up), 12 months (9-month follow-up), and 24 months (21-month follow-up). DatStat will send survey links and subsequent reminders via email/text according to the survey schedule. Study staff will also call and/or send text message reminders to participants to facilitate completion of online surveys as needed. All participants will continue to receive usual psychosocial care per institutional standards, as well as a standardized list of young adult-specific resources (see Enhanced Usual Psychosocial Care section). Usage of these services will be tracked via patient self-report at each survey. Participants will receive $25 for each of the first four surveys completed, and a $50 gift card for completing the final survey (up to $150 total).

Supplemental work (Administrative and extension)

A. Up to thirty young adults from our sample, who provided consent to be contacted, will be asked to participate in a qualitative interview at the 2-year follow-up, who are approximately two to three years post-enrollment, to understand how current survivorship care practices for young adults align with the newly established National Standards for Cancer Survivorship Care and identify barriers and facilitators to obtaining high-quality survivorship care.

B. To identify barriers and facilitators to implementing Bright IDEAS-YA in real-world clinical practice settings, we will analyze existing data from the session 6 audiorecordings from intervention participants and conduct qualitative interviews with up to 30 key informants (i.e., young adult survivors, community oncology providers, non-profit organization administrators). For the cultural/linguistic translation, eligible participants will be young adult cancer survivors

who were diagnosed with any cancer between the ages of 15 and 39 years, are within 3 years of diagnosis (to allow reflection on timing of intervention), identify as Hispanic, and speak Spanish and/or English. Up to 20 young adults will be recruited to participate in focus groups (3-4 consisting of 5-7 participants) or semi-structured interviews using Rutgers Zoom for feedback on current intervention and materials to identify strategies for tailoring. Semi-structured qualitative interviews and/or focus groups will last about an hour and participants will be paid $50 for their time and effort. Results will be used to revise Bright IDEAS-YA and translate it into Spanish. Following revision, we will conduct a small feasibility pilot of culturally/Linguistically tailored Bright IDEAS-YA. We will recruit 20 young adult cancer patients using the same criteria as the parent award (i.e., age 15-39, first diagnosis of any cancer within past 4 months, receiving chemotherapy, radiation, immunotherapy and/or stem cell transplant), with the exception that we will focus only on Hispanic young adults (English- or Spanish-speaking). We will recruit from the three recruiting sites of the parent award. They will be consented, asked to complete a baseline survey, complete the intervention with a trainer in their preferred language, then complete a follow-up survey. They will receive a $25 per survey for a total of $50.

B. Data Points

All participants will be asked to complete follow-up surveys at 3 months (post-intervention), 6 months (3-month follow-up), 12 months (9-month follow-up), and 24 months (21-month follow-up). Study staff will also collect data regarding participant completion of intervention sessions and trainer fidelity. Intervention sessions will be audio-recorded with participant permission for fidelity evaluation and trainer supervision.

For our Supplemental work (Administrative and extension), qualitative data will be collected in a one-time semi-structured interview or a focus group. For the small feasibility pilot of the culturally /linguistically tailored Bright IDEAS-YA, participants will be asked to complete a baseline and a post-intervention survey (at approximately 6-12 weeks).

C. Study Duration

The overall study will last 7 years. Each subject will participate for 2 years.
Qualitative interviews/Focus groups: Participants will be asked to complete a one-time interview or a focus group lasting approximately 60 minutes.
Culturally/Linguistically tailored feasibility pilot: participants will be asked to complete the intervention over a period of 6-12 weeks (6 weekly calls with a trainer).

D. Endpoints

The primary endpoint will be the estimated change from baseline to 6 months in depression, anxiety, and psychosocial HRQOL (i.e., Social/Family Well-Being and Emotional Well-Being), selected to allow for examination of temporal mediation of problem-solving ability post-intervention. The changes from baseline to 3 months, 12 months, and 24 months will be considered secondary endpoints.
Culturally/linguistically tailored pilot: endpoints are feasibility (enrollment, retention, acceptability, satisfaction).

1.4 Preliminary Data

The Bright IDEAS intervention has demonstrated effectiveness in increasing problem-solving skills, decreasing distress, and reducing symptoms of depression and post-traumatic stress of caregivers who are managing their child's cancer treatment.[20-22] We developed Bright IDEAS-Young Adults (Bright IDEAS-YA) to meet the needs of young adult patients during the critical months following a new cancer diagnosis. We used the Bright IDEAS acronym to remind participants of essential components of the intervention – fostering positive (vs. negative) problem orientation and using rational (vs. impulsive or avoidant) problem-solving skills. We completed a pilot study to gather data about the feasibility of using Bright IDEAS with AYA cancer patients (n=40). Analysis of the first 24 participants with complete data

showed that patients reported a high satisfaction with the intervention (*M* = 4.6 out of 5, *SD* = 0.5). As expected, patients used Bright IDEAS-YA to solve a variety of problems across life domains, including: (1) coping with the uncertainty of cancer; (2) deciding to go back to school; (3) returning to work/figuring out career; (4) feeling isolated from friends; (5) eating healthy/losing weight; (6) dating with cancer; and (7) challenges moving back home with parents. Qualitative feedback indicated that participants found Bright IDEAS-YA to be easy to learn and use, emphasizing the systematic approach and structure as most helpful. They noted that it was a skill they learned and can now use going forward. Young adults also reported that having a supportive trainer teach the skill was valuable.

Results were promising, with moderate to large effects on problem-solving ability (particularly improved positive problem-orientation and rational problem solving and reduced negative problem orientation and avoidant style). We found small to moderate improvements in depression, anxiety, and emotional health-related quality of life (HRQOL). We concluded that Bright IDEAS-YA is feasible and acceptable, with promising results that suggest efficacy testing is warranted.

1.5 Sample Size Justification
Up to 344 YA patients newly diagnosed with cancer will be enrolled across sites. There are no exclusion criteria based on gender or race. We expect the percentage of male/female and minority race participants enrolled to reflect the distributions seen at three sites.

Power focused on Aim 1 and was calculated based on effect size estimates that incorporate both estimates of change in mean as well as standard deviations from our pilot data and compared against published data, assuming an intent-to-treat analysis. For the proposed study, we assume the standard deviation will be 70% of the pilot standard deviation, due to tightening of the eligibility criteria to exclude those with cancer recurrence and recruit within 4 months of first diagnosis. In addition, we assume that the control group will see 20% of the change in depression compared to intervention. This leads to a Cohen effect size of 0.26. Assuming 344 subjects with 30% dropout, we will have 85% power to detect an effect of intervention on the Depression score. Similar calculations demonstrate 82% power to detect an effect of intervention on Anxiety. If we observe a group difference closer to 3.22 for Depression, cited as clinically meaningful in previous literature,[23] then under the assumptions listed above, our Cohen effect size will be closer to 0.29 and our power for the primary outcomes will be higher. For HRQOL, pilot Cohen estimates of effect size and estimates from the literature with the assumptions above would yield a Cohen effect size of 0.32, which would yield a power of 95%.

Supplemental work:
Aim 4 Individual interviews will be conducted with a subset (n=30) of young adults enrolled in the trial who agreed to future contact. We will purposefully sample 10 young adults from each site (i.e., Rutgers Cancer Institute, Memorial Sloan Kettering Cancer Center, and Moffitt Cancer Center). Target sample size is based on the literature and the research experience of our team, which show depth interviews within groups generally reach theoretical saturation by 6-10 interviews and that participants selected based on a fairly homogenous target population (i.e., YA survivors, ages 18-39) is optimal and considered robust. We will aim to recruit an equal number of participants by age (18-29 vs. 30-39) and site (n = 10 per site). Within site, we will purposefully recruit participants to achieve maximum variation among participants (i.e., racial/ethnic background, sex, work/school status, marital status). We will not set specific targets for these characteristics but ensure we have representation on these major factors.

Aim 5 Qualitative interviews: We will conduct up to 30 interviews with two main groups of key informants: young adult cancer survivors and providers/administrators in community settings (i.e., community hospitals or non-profit organizations serving young adult cancer survivors). As noted above, we anticipate saturation by 6-10 interview per group and we aim to recruit different types of healthcare providers from different organizations to achieve diverse views. For the cultural/linguistical adaptation work, we anticipate a total of 3-4 focus groups consisting of 5-7 participants (~15-20 total participants) will be adequate to achieve saturation of themes. Prior work has demonstrated saturation within 9-17 interviews or 4-8 focus groups.[24] We plan to run at least two groups in Spanish. However, we will continue to recruit

if saturation is not achieved. For the feasibility pilot of culturally/linguistically adapted Bright IDEAS-YA, will recruit 20 young adult cancer patients using the same criteria as the parent award, with the exception that we will focus on Spanish-speaking individuals who identify as Hispanic. This sample size is considered adequate for answering the feasibility questions posed.[25]

1.6 Study Variables

A. Independent Variables, Interventions, or Predictor Variables

Bright IDEAS-YA Intervention: Bright IDEAS-YA is a manualized problem-solving skills training intervention conducted by a trainer who teaches the participant the Bright IDEAS stepwise approach to problem-solving and guides the participant through solving their own problems using the Bright IDEAS approach and worksheets. The intervention consists of six 45-minute one-on-one sessions held at the clinic/hospital, by telephone between a patient and a trainer, or via video-conference session on HIPPA compliant Doxy.me (https://doxy.me/), or another institutionally-approved telehealth platform (e.g. Rutgers Zoom, site-specific telehealth platforms). Sessions are held weekly. However, due to the changing demands of cancer treatment and side effects that sometimes cause patients to feel too ill to do a session as scheduled, we allow up to 12 weeks to complete the 6 sessions. During the first face-to-face or virtual session, participants will be introduced to the Bright IDEAS model and the participant manual that explains the approach and provides worksheets for systematically solving problems. "Bright" refers to the sense of optimism that is critical to finding constructive solutions to difficult challenges. The letters in IDEAS represent the five essential steps of the PSST problem-solving approach (see Figure 1): "I"= "Identify the problem"; "D"="Determine the options"; "E"="Evaluate options and choose the best"; "A"="Act"; and "S"="See if it worked." Sessions 2-5 involve using the model to work through challenges identified by the participant. Session 6 involves a summary of the model, discussion of how the participant can continue to use the skills learned after the sessions end, and solicitation of feedback regarding the program and the website/app. See Table 1 for overview of each session.

Table 1. Overview of Bright IDEAS-YA Intervention Sessions

Session	Goals/Activities	Mapping to Problem-Solving Ability
**1**	1. Establish rapport<br>2. Explain general rationale for Bright IDEAS-YA approach<br>3. Review the 5 steps – Identify, Define your options, Evaluate options and choose, Act, See if it worked<br>4. Establish basic expectations for participation	- Reframes stressors as challenges that can be overcome, enhancing positive problem orientation and reducing negative problem orientation.<br>- Introduces 5-step approach, building rational problem-solving skills.
**2-5**	1. Review homework from last session<br>2. Work on a patient-identified problem using Bright IDEAS-YA worksheets<br>3. Identify homework for participant to use Bright IDEAS-YA between sessions	- Apply skills systematically to personal problems encourages self-efficacy to work through challenges (positive problem orientation), builds rational problem-solving skills, reduces impulsive responding, and prevents avoidance.<br>- Homework and follow-up reduce avoidance.
**6**	1. Review and reinforce the elements of Bright IDEAS-YA<br>2. Elicit feedback about experience using Bright IDEAS-YA<br>3. Review "relapse prevention" – continue to use skills instead of returning to prior problem-solving strategies<br>4. Thank patient for their participation	- Reviewing success fosters self-efficacy and positive problem orientation.<br>- Relapse prevention reinforces continued use of these skills to any challenge young adults encounter, including achieving normative life milestones.

Enhanced Usual Care Comparison Group: The Enhanced Usual Psychosocial Care group will complete surveys, receive the list of resources from the NCCN adolescent and young adult patient guildeline,[19] and receive usual psychosocial care per institutional standards. Usual psychosocial care generally consists of an initial social work consultation at the time of diagnosis, referral to necessary social resources such as transportation or financial assistance services according to patient needs, and optional supportive counseling or pastoral care if sought by the patient. Ongoing service use is determined by patient needs and preference. The standardized NCCN list of resources will be given to patients in both arms to control for awareness of young adult resources. Listed resources will include websites and organizations that provide informational (e.g., websites with information about young adult cancer treatment and side effects), emotional (e.g., young adult online support groups) and practical (e.g., transportation assistance, financial assistance) support services for young adults.

B. Dependent Variables or Outcome Measures

We will evaluate changes in the following outcomes: depression, anxiety, and health-related quality of life. See section 1.9B Study Instruments for details on each measure.

Supplemental Work: Semi-structured interview/focus group guides will be used. Outcomes will be qualitative reports of the extent to which survivorship care for young adults align with the National Standards for Cancer Survivorship care and barriers and facilitators to obtaining high quality survivorship care. For cultural adaptation: A semi-structured interview guide will be used to elicit input on the relevance of intervention materials and procedures, strategies for integrating cultural beliefs and values, preferred delivery methods, and important characteristics of interventionists with Hispanic young adult cancer survivors. The feasibility pilot outcomes include enrollment and retention rates, satisfaction and acceptability of the culturally/linguistically adapted Bright IDEAS-YA (using the same satisfaction measure as the parent trial listed in section 1.9B).

1.7 Drugs/Devices/Biologics

N/A

A. Drug/Device Accountability and Storage Methods

N/A

1.8 Specimen Collection

A. <u>Primary</u> Specimen Collection

N/A

B. <u>Secondary</u> Specimen Collection

N/A

1.9 Data Collection

A. <u>Primary</u> Data Collection

- **<u>Location</u>**: Surveys can be completed in a private space during a patient's routine treatment visit at each respective site or at home by the participant. The survey link will be emailed and/or texted to the participant, or a paper copy will be sent to their home address, if requested.
  <u>Supplemental work</u>: Qualitative interviews/focus groups will be conducted using Rutgers Zoom. The feasibility pilot will follow same procedures as the parent efficacy trial.
- **<u>Process of Data Collection</u>**: Surveys will be administered online using DatStat, a HIPAA-compliant electronic data capture system. For in-person administration, research staff will provide a tablet for patients to complete the online survey (paper copies will be available as

back-up in the event of significant internet disruption). For at-home completion, research staff will email or text the link to the survey to participants, or, if preferred, a paper copies will be mailed to them with a postage-paid return envelope.

- **Timing and Frequency**: Surveys will be administered five times – once at baseline, then again at 3 months (post-intervention), 6 months (3-month follow-up), 12 months (9-month follow-up), and 24 months (21-month follow-up). We anticipate each survey will take approximately 25 minutes to complete.
- **Procedures for Audio/Visual Recording**: Participants will be asked to give permission to audio record the intervention sessions for evaluation of treatment integrity and supervision of the trainers. As part of the consent process, participants will be told that it is completely voluntary to agree to the recording and they can change their mind at any time. The trainer will also ask the patient if it is okay to record at the beginning of each session. It will not be considered a protocol violation if technical issues prevent or disrupt recording or a trainer forgets to record a session. Digital recordings will be transferred off of the audio recorders as soon as possible following each session to the study folder on a cloud-based file storage application for a secure storage, management and sharing of digital files (e.g., Rutgers OneDrive, site-specific approved platform), and then erased from the recorder. Recordings will be labeled with a subject number rather than any identifiable information. Session audio recording files may be securely transferred between members of the research team using Rutgers secure large file transfer service (LiFT) or site-specific secure file transfer methods.

  Supplemental work: Participants will be asked for permission to audio record the interview/focus group using the recording feature of Rutgers Zoom. Digital recordings will be securely transferred and stored using Rutgers OneDrive. Professional transcription services will be used to transcribe all audio recordings, with the text files labeled with participant ID and any identifiable information removed from the transcript. For the feasibility pilot, participants will be asked to give permission to audio record intervention sessions for evaluation of treatment integrity and analysis of session discussions.

- **Study Instruments**:
  - Social Problem-Solving Inventory-Revised Short Form (SPSI-R:S)[26] is a 25-item self-report measure of five theoretically-important constructs of everyday problem-solving, including positive problem orientation, negative problem orientation, rational problem-solving style, impulsive/carelessness style, and avoidant style. Summary scores ranging from 0-20 are computed for each subscale, as well as an overall score. A higher Total score indicates better problem-solving ability. The SPSI-R:S has demonstrated strong reliability and validity estimates in our prior work[20,22,27] and other studies.[26]
  - PROMIS Depression Short Form (v1.0 8a) and PROMIS Anxiety Short Form (v1.0 8a)[28] are 8-item measures of depressive symptoms and anxiety from the NIH Patient-Reported Outcomes Measurement Information System (PROMIS).[29,30] Respondents report symptoms on a 5-point rating scale from 1 (*never*) to 5 (*always*) in the past 7 days, with higher scores indicating higher levels of negative affect. These measures are scored using the online Assessment Center, which calculates a total raw score and translates it into a standardized T-score with a mean of 50 and standard deviation of 10. Depression items focus on affective and cognitive symptoms of depression (sadness, worthlessness, loss of interest) rather than somatic symptoms (change in sleep or appetite) to avoid potential confounding with medical conditions. The Anxiety scale focuses on fear (panic), anxious misery (worry, dread), and hyperarousal (tension, nervousness). These measures have demonstrated clinical validity and responsiveness to change.[31,32]

- The Functional Assessment of Cancer Therapy – General (FACT-G v4)[33] is a 27-item widely used and well-validated measure of health-related quality of life for adult cancer patients ages 18 and older. It yields an overall General Total score and four subscales: Physical Well-Being (PWB), Social/Family Well-Being (SWB), Emotional Well-Being (EWB) and Functional Well-Being (FWB). Respondents rate each item on 5-point Likert scale from 0 (*not at all)* to 4 (*very much)* in the past 7 days. Each scale is a summary of responses, ranging from 0 to 28 for PWB, SWB, and FWB, 0 to 24 for EWB, and 0 to 108 for General Total.
- Financial strain, a marker of socioeconomic status particularly relevant for young adults with cancer, will be measured using the Comprehensive Score for Financial Toxicity (COST),[34] an 11-item scale of financial toxicity associated with cancer treatment. Respondents rate each item on a 5-point Likert scale from 0 (*not at all)* to 4 (*very much)* in the past 7 days. The measure yields a total summary score, with higher scores indicating greater financial strain. It has demonstrated adequate internal reliability and face validity.[34] These items will be administered at baseline, 12 months, and 24 months. Given that young adults may demonstrate different levels of financial independence, those who indicate that they are not primarily responsible for paying for their medical care will respond to only two items regarding financial stress at baseline only. Those who indicate financial responsibility at baseline and everybody at 12 months, and 24 months follow-up, will respond to the full 11-item scale.
- Unmet needs and concerns will be measured using the Adolescent and Young Adult Oncology Screening Tool, which is an adapted version of the NCCN Distress Thermometer and problem checklist to include problems specific to young adults.[35] Participants first rate their distress on a 0-10 scale. Next, they are asked to check off areas of concern from the past week, including practical (e.g., housing arrangements, work, bills, transportation), family (e.g., parents, siblings, partner), emotional (e.g., sadness, isolation, guilt), social (e.g., isolation from friends, missing important events), physical (e.g., body image, sexual concerns, sleeping difficulty), and informational (e.g., understanding information, feeling involved in decision-making) needs. A total score from 0 to 51 is calculated. This checklist is used to prompt selection of problems to be addressed in the intervention for those assigned to receive the intervention.
- Demographic/Medical: Patients will report age, sex, independent living status, race, ethnicity, school/work status, health insurance coverage, marital status, whether he/she is a parent, and income. Potentially time-varying characteristics (i.e., school/work status, insurance status, income) will be repeatedly assessed at all follow-up surveys. Cancer diagnosis, date of diagnosis, and treatment received (e.g., chemotherapy, radiation, hematopoietic stem cell transplant), will be abstracted from the medical record by research staff. This information will be used to describe the sample and used as covariates in the model (except for sex, which will be evaluated as a moderator). Observational research with adolescents and young adults found being on treatment and not being in school or working to be the primary drivers of distress rather than age, race, or cancer type.[36]
- Supportive Services Received will be measured via self-report where participants will check whether they have used or received services in the following categories: psychosocial support, informational and practical support, fertility or sexual health, physical/wellness services and integrative medicine services) since diagnosis (at baseline) or the prior survey (at 3, 6, 12, and 24 months). If they check yes, they will be asked to specify the service used.
- PROMIS Social Isolation –Short Form 4a is a four-item measure assessing perceptions of social isolation from the NIH Patient-Reported Outcomes Measurement Information System (PROMIS.) [29,30] Each question uses a 5-point rating scale from 1 (*never*) to 5 (*always*) in the past 7 days, with higher scores indicating higher levels of perceived isolation.

- o <u>Participant Satisfaction</u> will be assessed using a 10-item questionnaire derived from the Multi-Dimensional Treatment Satisfaction Meausre[37] to assess utility of intervention-specific components (e.g., the user manual, worksheets), attitude towards the intervention, trainer competence, and perceived benefit attributable to the intervention, plus 3open-ended questions eliciting ideas for improvements. This questionnaire will only be administered at post-intervention to intervention arm only (3 months).
- o <u>Coronavirus Impact Scale</u>[38] is a 12-item scale to assess how COVID-19 has changed a person's life (e.g., routine, family income, food access, medical and mental health access, access to family and friends, etc.) This will be administered at baseline.
- o <u>Treatment Integrity (TI) Checklists</u> will be completed by a qualified member of the research team from any site. The reviewer will listen to the randomly selected session audio recording and complete a structured checklist specific to the manualized content of each session (separate checklists for session 1, 2-5, and 6).[22] Items rate the quality of therapeutic alliance (e.g., "trainer and participant clearly engaged, with good rapport, working together throughout session"), communication (e.g., "trainer uses a variety of techniques to clearly communicate that young adult is being listened to throughout session"), and fidelity to the treatment manual (e.g., "session includes application of Bright IDEAS-YA steps to a participant problem," "trainer offers 3 or more strategies for relapse prevention").
- o **<u>Internal Tracking</u>** (completed by research team)
  - ▪ <u>Eligibility Checklist:</u> This checklist will verify eligibility and document reasons for non-participation.
  - ▪ <u>Participant Progress Tracking:</u> The Research Assistant at each site will be responsible for registering and tracking all participants at their site using DatStat, a HIPAA-compliant cloud-based software used for participant tracking, study flow, and administration of patient surveys. Each relying site will only have access to their own participant data to maintain confidentiality. DatStat automates study workflow to remind Research Assistants of upcoming or overdue study tasks (such as tracking of session and survey completion) and can directly email participants links to complete surveys and reminders according to the study schedule. This system also tracks participant incentives. DatStat also produces study flow charts to monitor recruitment and retention throughout the study.

- ▪ **<u>Ethnographic Studies, Interviews, Or Observation</u>**: For the supplemental work, semi-structured interview/focus group guides will be used to conduct the interviews.
- ▪ **<u>Subject Identifiers</u>**: We will collect participant name, address, telephone number, and email address for contact purposes throughout the study. Each participant will be assigned a participant ID number and we will keep a key linking the ID number to name for up to 6 years after the close of the study. We will collect MRN, date of birth, and date of diagnosis to abstract medical treatment data, calculate age, and calculate time since diagnosis. At the conclusion of the study, identifiers will be removed from the database and de-identified data kept indefinitely.

B. <u>Secondary</u> Data Collection

- ▪ **<u>Type of Records</u>**: Medical records will be accessed to abstract: date of birth, date of diagnosis, type of diagnosis to determine eligibility and describe the study sample. At the last survey follow-up, records will be accessed to document treatment(s) received, relapse status, comorbid psychological conditions, any documented social concerns, hospice referral, COVID-19 vaccination history, and use of psychosocial services. For those subjects who do not remain in the study until last follow-up due to various reasons (e.g., no longer interested, deceased, etc), data abstraction will occur at time subjects are exited from the study or as soon as practical.

- **Location**: Research staff with authorization to access medical records will access the medical records at each site using an authorized workstation to access the record.
- **Inclusion/Exclusion**: See Inclusion/Exclusion criteria in section 4.1C.
- **Data Abstraction Form(s):** See Data Abstraction Form.

1.10 Timetable/Schedule of Events

Arm	Baseline Survey & Randomization	Bright IDEAS-YA Sessions	3-month Survey	6-month Survey	12-month Survey	24-month Survey
Bright IDEAS-YA Intervention	X	X	X	X	X	X
Enhanced Usual Care Comparison	X		X	X	X	X

2.0 Project Management

2.1 Research Staff and Qualifications

The PI and research staff at each site will have the appropriate qualifications and training to conduct the study at each site.

Dr. Devine, the overall study PI, oversees the entire project. She is an expert trainer in Bright IDEAS and has trained other qualified individuals (e.g., graduate students, social workers, psychologists) to conduct the intervention. She will train and supervise all research assistants who will deliver the Bright IDEAS-YA intervention. All research assistants who will serve as trainers will be added via modification and approved as study staff in the IRB protocol prior to any patient contact. Each trainer will undergo a standardized Bright IDEAS training led by Dr. Devine. This training includes education about the foundations of the intervention, reviewing the instructor's manual with guidelines for each session, viewing videotaped training videos, and role-playing intervention sessions. Trainers do not have to be professional counselors but are required to complete this training prior to working with any participant. Trainers will also complete CITI, HIPAA, and other required research trainings.

Kristine Levonyan-Radloff, the project coordinator, has significant experience managing research studies and will meet at least weekly with Dr. Devine.

Dr. Denalee O'Malley is a mixed method implementation science researcher with expertise in qualitative data collection, multi-level stakeholder engaged research, and cancer health disparities. She will lead the administrative supplement qualitative work, including developing and finalizing the qualitative interview guide, training and supervising the research assistants who will conduct the interviews, and overseeing the data analyses.

Dr. Meredith Collins is a health communications researcher with significant experience in conducting and analyzing in-depth interviews with adolescent and young adult cancer survivors. She will assist Dr. O'Malley in developing and finalizing the qualitative interview guide, conduct some of the interviews, and participate in the coding team.

Dr. Jenna Howard is a consultant with expertise in qualitative research and analysis. She will assist in conducting some of the interviews and analyzing/interpreting the data.

Dr. Evelyn Arana, a co-investigator with expertise in culturally and linguistically adapting behavioral interventions for adult cancer survivors, will lead the cultural adaptation process.

2.2 Research Staff Training

All staff is trained and compliant with CITI requirements to conduct research. Additionally, all staff will attend study start-up meetings to review the protocol, become familiar with study procedures and the proper conduct of the protocol.

2.3 Resources Available

All participants will be patients undergoing treatment at their respective cancer centers and will have access to usual psychosocial care as needed (e.g., social work and/or psychologist). All Site PIs are licensed psychologists who can assist patients in obtaining referrals as needed.

At the Rutgers Cancer Institute of New Jersey, participants will have access to social work and other psychological resources as needed. Dr. Devine is a psychologist and can make referrals for psychological resources if necessary.

2.4 Research Sites

Participant recruitment will take place at:

1) Rutgers Cancer Institute of New Jersey, New Brunswick, NJ
2) Memorial Sloan Kettering Cancer Center, New York, NY
3) Moffitt Cancer Center, Tampa, FL

IRB reliance agreements will be obtained and uploaded prior to engaging in research.

The University of Rochester will contribute intellectually to the research and access coded audio recordings to conduct treatment fidelity ratings but will not store any data or have access to any PHI or the code linking data and subject identifiers. A letter of cooperation is uploaded.

3.0 Multi-Center Research

Rutgers will serve as the IRB of Record for this multisite study. IRB reliance agreements will be obtained and uploaded prior to engaging in research.

Communication Plan. At the beginning of the project, Dr. Devine will host a three-hour virtual kick-off meeting using Webex videoconference to plan each site's action steps to initiate the project (i.e., finalization of protocol and timeline for regulatory submissions and hiring staff). Once each site has hired the personnel who will serve as Bright IDEAS-YA trainers, Site PIs, project coordinators, and interventionists will attend a 1.5-day in-person or remote training at Rutgers with Dr. Devine. The workshop training has been standardized based on prior Bright IDEAS clinical trials and the dissemination trial led by Co-I Sahler that taught mental health professionals how to use Bright IDEAS (R25CA183725). It involves didactic presentation of the skills needed to teach Bright IDEAS and the goals of each session, live and video-recorded demonstrations of these skills, and role playing with constructive feedback. Site PIs will conduct future trainings using this standardized approach for additional personnel as needed, with Dr. Devine available via videoconference to answer questions and provide feedback. All interventionists will be supervised regularly throughout the trial (See Treatment Integrity section). The PI will coordinate monthly phone calls with all co-investigators and study staff throughout the project to discuss enrollment, retention, data management, and implementation issues. Dr. Devine and the project coordinator will also hold weekly calls with site research staff to ensure compliance with study procedures and accrual targets.

4.0 Subject Considerations

4.1 Subject Selection and Enrollment Considerations

A. Method to Identify Potential Subjects

- RCT: At each site, patients will be identified through electronic medical record review of active patients and by referrals from treating physicians/nurses/social workers. Research study staff will contact treating physicians or other qualified treatment team member

familiar with the patients, to assess medical eligibility of patients. If a physician or other treatment team member feels their patient is not suitable for participation, we will not approach the patient. Patients who pass initial screens will be approached in person by research staff during a routine medical visit or contacted by letter/phone or email (if available).

- Administrative Supplement: Individual interviews will be conducted with a subset (N=30) of young adults enrolled in the RCT. We will purposively sample 10 young adults from each site. We will aim to recruit an equal number of participants by age (18-29 vs. 30-39) and site (n = 10 per site). Within site, we will purposefully recruit participants to achieve maximum variation among participants (i.e., racial/ethnic background, sex, work/school status, marital status). We will not set specific targets for these characteristics but ensure we have representation on these major factors.
- Extension Supplement: Key informants (young adult cancer survivors and providers/administrators in community settings) will be recruited for individual interviews. Survivors will be recruited from the pool of young adult survivors from the parent trial who agreed for future contact and via advertisements on social media, non-profit organizations, and provider referral. We will use a combination of professional networks and public information to identify providers/administrators. Young adult cancer survivors who identify as Hispanic for the cultural/linguistic adaptation of the Bright IDEAS work will be recruited to participate in a focus group or a semi-structured interview and the feasibility pilot from the three recruiting sites. We will explore social media and other methods of recruitment if needed.

B. Recruitment Details

RCT: Trained research staff will approach potentially eligible patients during a routine medical visit or via letter/phone, or e-mail (if available). If in-person, study staff will hand out the study information sheet and flyer, explain the study, assess interest/eligibility, allow adequate time for review and discussion with family if needed, and obtain informed consent if patient is eligible and interested. If by letter/phone, study staff will mail study cover letter along with the study information sheet and flyer to explain the study and let them know they will be contacted by phone by a member of the study team to assess interest and eligibility. If patient's email address is available, an email will be sent to introduce the study and to let them know the study staff will follow-up with a phone call. Staff will be rigorously trained as to how to approach patients, introduce the study, communicate about confidentiality and voluntary participation, and engage in the process of informed consent in a culturally-competent way.[39] Recruitment scripts, cover letter, information sheet, e-mail wording and flyer are being uploaded for approval.

For the qualitative supplemental work, research study staff will reach out to survivors and providers to invite them to participate in an individual qualitative interview and/or focus group via phone, email, or in-person, when feasible. For the feasibility pilot, recruitment will follow the same approach as the parent RCT.

C. Subject Screening

A study staff member will screen interested patients during their initial contact. Study staff member will approach potentially eligible patients (i.e., within the age range and with initial physician/nurse/social worker's recommendation) in clinic, send a recruitment letter/email, or call, if not scheduled for a visit, using the Recruitment Script to explain the study and answer any questions from potential participants. If the patient is interested, study staff member will administer the Eligibility Checklist. If patient is eligible and willing to enroll in the study, the study staff member will continue the Consent process.

- **Inclusion Criteria (RCT)**

  Participants will be eligible if:

  1) Current age 18-39 years

2) Within 4 months of first* diagnosis of any cancer (*patients diagnosed previously with non-melanoma type of skin cancer treated with surgery only may also be included).
3) Cancer is being treated with chemotherapy and/or radiation therapy and/or hematopoietic stem cell transplant (immunotherapy included)
4) No documented or self-reported cognitive delay or impairment that would prevent completion of survey measures
5) English-speaking

- Inclusion Criteria for the cultural/linguistic adaptation qualitative work include:
    1) Current age 18-39 years
    2) Diagnosed with any cancer between the ages of 15 and 39
    3) Within 3 years of diagnosis (to allow reflection on timing of intervention)
    4) Identify as Hispanic
    5) Speak Spanish and/or English.
- Inclusion Criteria for the pilot feasibility: Same as RCT but must identify as Hispanic and speak English or Spanish.

- **Exclusion Criteria**
    1) Life expectancy < 6 months per physician/treatment team report
    2) Treatment involves surgery only

4.2 Secondary Subjects

N/A

4.3 Number of Subjects

A. Total Number of Subjects

RCT: 344
Administrative Supplement: Subjects will be part of the original 344 enrolled and will provide additional consent for the interviews
Extension Supplement:
Qualitative interviews: Up to 30
Cultural adaptation interviews/focus groups: Up to 20
Feasibility pilot: 20
Total inclusive of RCT and supplemental work: 414

B. Total Number of Subjects If Multicenter Study

RCT: 344
Supplemental work: 70
Total: 414

C. Feasibility

Each site has a large enough patient population to support recruitment of the target number of participants. Combined across sites, the estimated patient pool is 3,450 over 3 years; acceptance rates would have to fall below 10% to not meet accrual goals, which is highly unlikely given our acceptance rate of 66% in our pilot study.

4.4 Consent Procedures

A. Consent Process

- **Location of Consent Process**
  RCT: The consent process will take place during a patient's routine medical visit (in-person or telehealth), or via phone.

Administrative Supplement: Consent will take place over the phone or Zoom.
Cultural adaptation interviews/focus groups: Consent will take place over the phone or Zoom.
Feasibility pilot: Consent will take place during a patient's routine medical visit or via phone, as in parent RCT.

- **Ongoing Consent**
  The trainers conducting the intervention sessions will also be trained to check in with participants at each session their ongoing willingness to participate in the study. Each follow-up survey will begin with a paragraph of instructions reminding participants of the voluntary nature of their participation.
  Administrative Supplement/Cultural adaptation: N/A – single interview/focus group session
- **Individual Roles for Researchers Involved in Consent**
  The PI, project coordinator, or trained research staff member will obtain consent.
- **Consent Discussion Duration**
  We expect most initial consent discussions to take approximately 10 minutes. The trainers conducting the intervention sessions will also be trained to check in with participants at each session about their ongoing willingness to participate in the study.
- **Coercion or Undue Influence**
  The PI or trained research team member obtaining consent is not a part of the treating team and will clearly state that participation is voluntary and the decision to take part in the study or not will have no effect on the person's ongoing treatment or relationship with their treating team.
- **Subject Understanding**
  The PI or trained research team member obtaining consent will encourage questions and ask the participant to state in their own words their understanding of the research study, including risks, benefits, and voluntary nature of participation. RCT and feasibility pilot consent form will contain a few questions to assess understanding.

B. Waiver or Alteration of Consent Process

- **Waiver or Alteration Details**
  N/A
- **Destruction of Identifiers**
  N/A
- **Use of Deception/Concealment**
  N/A
  a. **Minimal Risk Justification**
     N/A
  b. **Alternatives**
     N/A
  c. **Subject Debriefing**
     N/A

C. Documentation of Consent

- **Documenting Consent**
  RCT: Informed consent will be obtained from every participant prior to beginning the study via online DatStat consenting (with electronic signature). In the event of internet disruption or technical difficulties, paper consent can be used. Signed consent forms can be securely downloaded from DatStat and stored in Rutgers BOX or as indicated in site-specific local context procedures.
  Feasibility pilot: Informed consent with electronic signature will be obtained following the same procedures as the parent RCT.

- **Waiver of Documentation Of Consent (i.e., will not obtain subject's signature)**
  RCT: N/A

Administrative Supplement: Verbal consent will be obtained prior to starting the interview or focus group. A copy of the consent form will be emailed to the participant. The staff member will document the participants' agreement but we will not obtain an electronic signature from participants.
Cultural adaptation interviews/focus groups: Verbal consent will be obtained prior to starting the interview or focus group. A copy of the consent form will be emailed to the participant. The staff member will document the participants' agreement, but we will not obtain an electronic signature from participants.
Feasibility pilot: N/A

4.5 Special Consent/Populations

A. Minors-Subjects Who Are Not Yet Adults

- **Parental Permission**
  N/A
- **Non-Parental Permission**
  N/A
- **Assent Process**
  N/A
- **Documentation of Assent**
  N/A
- **Reaching Age of Majority During Study**
  N/A

B. Wards of the State

N/A

- **Research Outside of NJ Involving Minors**
  N/A

C. Non-English-Speaking Subjects

N/A

- **Process for Non-English-Speaking Subjects**
  N/A
- **Short Form Consent for Non-English Speakers**
  N/A

D. Adults Unable to Consent / Decisionally Impaired Adults

N/A

- **NJ Law-Assessment of Regaining the Capacity to Consent**
  N/A
- **Capacity to Consent**
  N/A
  a. **NJ Law-Selecting A Witness**
  N/A
  b. **Removing a Subject**
  N/A

4.6 Economic Burden and/or Compensation for Subjects

A. Expenses

There are no costs associated with this study.

B. Compensation/Incentives

Participants will receive a $25 gift card for each of the first four completed surveys, and a $50 gift card for completing the final survey, up to $150 total. Small token gifts, such as Bright IDEAS stress balls or sticky pads, will be sent as a thank you for participants' time and effort to promote retention in the study. Furthermore, to promote retention, study staff will mail yearly holiday cards to subjects on study.

Administrative supplement: Participants will receive a $50 gift card for their time and effort.

Extension supplement: Participants in the qualitative interviews/focus groups will receive a $50 gift card for their time and effort. Participants in the pilot feasibility will receive a $25 gift card for completing each survey, for a total of $50.

C. Compensation Documentation

Research staff will maintain documentation of compensation in DatStat, which keeps records of the compensation sent electronically to participants, or a file on Rutgers OneDrive.

4.7 Risks of Harm/Potential for Benefits to Subjects

A. Description of Risks of Harm to Subjects

- **Reasonably Foreseeable Risks of Harm**
  This study involves minimal risk. There are no physical risks or side effects associated with the study. Potential emotional risks of study participation include possible discomfort or distress associated with answering survey questions about current emotional functioning, problem-solving skills, financial strain, and health-related quality of life, or discussing personal challenges during problem-solving sessions. Attending in-person sessions may be seen as an inconvenience, but virtual visits are available and the trainer attempts to minimize burden by scheduling when the participant is already scheduled for a medical visit or offering virtual sessions as needed. There is also a risk of breach of confidentiality of data provided for research, but safeguards are in place to minimize risks (see section E. Minimizing Risks below). Based on our prior experience conducting research with cancer caregivers, the likelihood that participants in this study will experience such discomfort or distress is low. The risks of harm for the qualitative supplemental work are minimal, including potential discomfort in answering interview or focus group questions or breach of confidentiality. Safeguards are in place to minimize these risks.
- **Risk of Harm from an Intervention on a Subject with an Existing Condition**
  All participants will be undergoing cancer treatment and the intervention is targeted to address the needs of these individuals. Individuals with a concurrent or past mental health diagnosis are eligible to participate and trainers or the PI can make referrals for additional psychological treatment resources as needed if participants present with mental health concerns.
- **Other Foreseeable Risks of Harm**
  A possible loss of confidentiality could cause embarrassment for a participant but unlikely to cause any social harm or consequences given no collection of sensitive data.
- **Observation and Sensitive Information**
  N/A

B. Procedures which Risk Harm to Embryo, Fetus, and/or Pregnant Subjects

N/A

C. Risks of Harm to Non-Subjects

N/A

D. Assessment of Social Behavior Considerations

Participant distress will be systematically monitored in several ways. First, the study coordinator at each site will review patient reported distress on the baseline survey. Individuals who report elevated distress scores (i.e., $\geq$8 on 0-10 scale) will be assessed for safety, and if they express no intent to harm themselves, will be referred to clinical social work services. If there is concern about participant safety in the medical setting, the study will alert the treating clinical staff to initiate patient

safety protocols and notify the site PI (licensed psychologist). If the participant is not at the medical center, research staff will coach them to go to their nearest emergency room for help and call 911 if needed. Second, interventionists will be trained to respond to patient distress at the time it occurs during intervention sessions, calling the Site PI (licensed psychologist) and referring to clinical social work services if needed. Third, study coordinators will review patient-reported distress at all subsequent surveys, intervening as above if elevated distress occurs and documenting procedures.

E. Minimizing Risks of Harm

Participants will be encouraged to contact the research team if they experience emotional discomfort or distress at any time during the study. All research staff will receive training to appropriately identify and handle instances when a patient would benefit from a referral (e.g., to a clinical social worker) for follow-up of emotional discomfort or distress.

To protect the confidentiality of participants, a series of security procedures will be undertaken. IRB and HIPAA regulations concerning confidentiality will be strictly enforced. All study personnel receive training and certification in human subjects protection and HIPAA regulations. Each study participant will be given a unique numeric identifier upon study entry. Names and other identifiable protected health information will not be stored in the same database as survey and medical information. All computers used for research purposes adhere to the institution's requirements regarding password protection, data encryption, anti-virus protection, and intrusion detection. All Internet-based data communications will be encrypted. This includes transfer of electronic audio files between approved members of the research team for treatment fidelity and supervision purposes if needed. All hard copy study-related materials and data will be stored in locked file cabinets in the site PI's locked office.

- **Certificate of Confidentiality**
  This study is NIH-funded and therefore a Certificate of Confidentiality is automatically issued to protect the data collected.
- **Provisions to Protect the Privacy Interests of Subjects**
  Participants will only be approached by trained and authorized research staff. Physicians or other appropriate treatment team members will be contacted for permission to approach prospective participants that study staff deem eligible based on the inclusion criteria. If anyone feels their patient is not suitable for participation, we will not approach the participant.

F. Potential Benefits to Subjects

Participants randomized to the Bright IDEAS-YA intervention may benefit by improving problem-solving skills and reducing symptoms of anxiety, depression, and distress. Prior studies with caregivers of pediatric patients have demonstrated such benefits[20-22] but it is unknown if AYA would benefit from this training. Participants randomized to the Enhanced Usual Care arm may or may not benefit from obtaining the list of resources from the NCCN adolescent and young adult patient guideline.
Supplemental work: There are no direct benefits to participants.

5.0 Special Considerations

5.1 Health Insurance Portability and Accountability Act (HIPAA)

HIPAA language is included in the consent form. As part of this study the following identifiable and/or protected health information will be collected: name, address, phone number, date of birth, email address, date of diagnosis, and IP address (surveys completed on DatStat collect this data but IP address will not be downloaded with the survey data). We will not be disclosing individually identifiable health information unless required by law.

5.2 Family Educational Rights and Privacy Act (FERPA)

N/A

5.3 Code of Federal Regulations Title 45 Part 46 (Vulnerable Populations)

A. Special Populations

- Prisoners: N/A
- Neonates: N/A
- Neonates of Uncertain Viability: N/A
- Children: N/A
- Individuals with Impaired Decision-Making Capacity: N/A

5.4 General Data Protection Regulation (GDPR)

N/A

5.5 NJ Access to Medical Research Act (Surrogate Consent)

N/A

6.0 Data Management Plan

6.1 Data Analysis

Aim 1: Efficacy of the Bright IDEAS-YA intervention vs. enhanced usual psychosocial care will be evaluated assuming repeated measures linear models, using maximum likelihood estimation (MLE) (SAS Proc Mixed).[40] All randomized participants, regardless of the extent to which they completed the intervention, will be included in intent-to-treat analysis. The benefits of this approach include: (1) all available data can be included in analyses; (2) correlation between related measures and adjusted test statistics can be estimated; (3) time varying covariates (such as school/employment status, treatment status (on treatment vs. completed), utilization of usual psychosocial care) can be incorporated into the model; and, (4) the assumptions about missing data from Missing Completely at Random (MCAR) to Missing at Random (MAR) are relaxed. Site will be included as a fixed effect. The primary hypothesis is that participants in Bright IDEAS-YA will experience greater decreases in depression and anxiety and increases in HRQOL at the 6-month follow-up relative to participants in the enhanced usual psychosocial care condition. The primary endpoint will be the estimated change from baseline to 6 months in depression, anxiety, and psychosocial HRQOL (i.e., Social/Family Well-Being and Emotional Well-Being), calculated as linear contrasts of regression parameters using the main effect of time and the interaction between time and treatment group from the mixed linear model. The changes from baseline to 3 months, 12 months, and 24 months will be considered secondary endpoints. For HRQOL, we are particularly interested in 12-and 24-month follow-ups given that changes may take longer to accrue compared with distress symptoms. Sensitivity analyses will be performed to determine the robustness of conclusions.[41] Planned sensitivity analyses include: (1) analysis with and without adjusting for site; (2) per protocol analysis (excluding participants who did not complete at least 4 out of 6 intervention sessions, selected because it is expected that participants would have adequate exposure and practice with the Bright IDEAS model by four sessions); (3) adjustment for imbalance in baseline characteristics, if applicable; and (4) analysis to examine any differences by racial/ethnic subgroups.

Aim 2: Mediation analysis will be used to evaluate problem-solving ability as a mediating variable between the intervention and each of the outcomes while also allowing for a direct effect of intervention on outcome. Specifically, bootstrap approaches will estimate the proportions attributed to the direct effect and to the effect mediated through problem-solving using the R program package mediation.[42]

A small direct effect is expected due to a positive therapeutic effect of talking to the trainer. Problem-solving ability will be measured by the total scale score and five subscales (positive problem orientation, negative problem orientation, rational problem solving, impulsive-careless style, and avoidant style), which will each be considered individually as potential mediators.

Aim 3: Potential moderators, including sex, financial strain, baseline unmet needs, and baseline distress, will be tested individually in models including all possible interaction terms. We will allow for each moderator to interact with time, arm, and time x arm to determine if it modifies the relationship between intervention and outcomes. We consider this analysis exploratory to identify subgroups who may benefit differentially from this intervention. We expect that females and participants with greater financial strain, baseline unmet needs, and baseline distress will benefit more.

6.2 Data Security

Only authorized study personnel will have access to study data, and access to identifiable data will be limited to staff who need the data to interact with participants. Survey and medical data will be in a separate database from identifiable data (e.g., name, address, etc.). The link between subject name and ID will be destroyed 6 years after the close of the study; other study data will be kept indefinitely. All computers used for research purposes adhere to the institution's requirements regarding password protection, data encryption, anti-virus protection, and intrusion detection. All Internet-based data communications will be encrypted. Physical records will be stored in a locked filing cabinet with the PI's locked office at each site. For participants who consent to be contacted for future studies (to be requested at the end of the final survey at 24 month follow-up), the study team will store their contact information (i.e., first and last names, phone number, email address) as well as cancer history (i.e., diagnosis, date of diagnosis, and end of treatment date) separately from research data on the PI's secure One Drive, with access granted only to the PI, study coordinator, and limited authorized research staff. We will store this information for up to 10 years from date of consent, after which it will be destroyed by the Primary Investigator.

6.3 Data and Safety Monitoring

A. Data/Safety Monitoring Plan

This is a minimal risk study. The PI and study staff at each site will continuously monitor for any potential adverse events (AEs). Anticipated adverse events include participant distress related to the intervention sessions or breach of confidentiality. Participant distress will be monitored in several ways. First, the study coordinator at each site will review patient reported distress on the baseline survey, which is generally completed in person at the same time as a routine medical visit. Individuals who report elevated distress (i.e., score ≥8 on 0-10 scale) will be assessed for safety, and if they express no intent to harm themselves, will be referred to clinical social work services. If there is concern about participant safety in the medical setting, the study will alert the treating clinical staff to initiate patient safety protocols and notify the site PI (licensed psychologist). If the participant is not at the medical center, research staff will coach them to go to their nearest emergency room for help and call 911 if needed. Second, interventionists will be trained to respond to patient distress at the time it occurs during intervention sessions, calling the Site PI (licensed psychologist) and referring to clinical social work services if needed. Third, study coordinators will review patient-reported distress as soon as possible following completion of all subsequent surveys, calling the patient to assess for safety as above if elevated distress occurs, making appropriate referrals, and documenting procedures. The online survey will also contain contact information for the study coordinator if patients have any questions or concerns while completing the survey. All staff will be trained to maintain confidentiality of patient data. Study staff will report any AEs immediately to the PI. Dr. Devine and the investigative team, in consultation with the Data and Safety Monitoring Board, will be responsible for evaluating each AE, determining whether it is study-related, and assessing any change to the risk/benefit ratio.

All unexpected and/or serious adverse events (AEs) occurring during the active portion of the intervention or up to 30 days after the last intervention session will be reported to the RBHS IRB in accordance with IRB policy. The research team will review and discuss via teleconference any serious adverse events to ensure protections to the participants and discuss any modifications to the protocol. If modifications are needed, they will be submitted immediately according to standard

IRB procedures. The PI will also conduct a quarterly review of safety and adverse events to be discussed during monthly all-investigator calls.

B. Data/Safety Monitoring Board Details
The PI will convene a Data and Safety Monitoring Board (DSMB) composed of a biostatistician, a behavioral scientist, and a physician who are not associated with the study. The committee will be responsible for oversight of patient safety and will meet yearly to review patient safety and trial progress. This review will include for each arm of the study: the number of patients enrolled, withdrawals, serious adverse events both expected and unexpected, and responses observed. A report with recommendations from the DSMB will be submitted annually to the RBHS IRB with the continuing review for the study.

6.4 Reporting Results

A. Individual Subjects' Results
N/A – results will be calculated on the aggregate data; no relevant data for sharing with subjects.

B. Aggregate Results
We will post aggregate results on clinicaltrials.gov for public viewing.

C. Professional Reporting
It is expected that the results of this research will be submitted for publication in a timely manner following the conclusion of the study. The PI and all co-authors must review any abstract or manuscript prior to submission.

D. Clinical Trials Registration, Results Reporting and Consent Posting
This trial will be registered at clinicaltrials.gov. Results will be reported and the approved consent form uploaded.

6.5 Secondary Use of the Data

We will make de-identified data available for sharing with other qualified researchers for secondary research. This is described in the consent form.

7.0 Research Repositories – Specimens and/or Data

N/A

8.0 Approvals/Authorizations

Rutgers will serve as the IRB of record for this study. Reliance agreements with the IRBs of participating sights will be obtained prior to commencing the research.

9.0 Bibliography

References

1. Bleyer WA. Cancer in older adolescents and young adults: epidemiology, diagnosis, treatment, survival, and importance of clinical trials. *Medical and Pediatric Oncology: The Official Journal of SIOP—International Society of Pediatric Oncology (Societé Internationale d'Oncologie Pédiatrique*. 2002;38(1):1-10.
2. Devins GM, Bezjak A, Mah K, Loblaw DA, Gotowiec AP. Context moderates illness-induced lifestyle disruptions across life domains: a test of the illness intrusiveness theoretical framework in six common cancers. *Psycho-Oncology*. 2006;15(3):221-233.
3. Smith AW, Bellizzi KM, Keegan THM, et al. Health-Related Quality of Life of Adolescent and Young Adult Patients With Cancer in the United States: The Adolescent and Young Adult Health

Outcomes and Patient Experience Study. *Journal of Clinical Oncology*. May 6, 2013 2013;doi:10.1200/jco.2012.47.3173
4. Zebrack BJ. Psychological, social, and behavioral issues for young adults with cancer. *Cancer*. 2011;117(S10):2289-2294.
5. D'Zurilla TJ, Nezu AM. *Problem-solving therapy: A positive approach to clinical intervention*. 3rd ed. Springer Publishing Company; 2007.
6. Chan RJ, Crawford-Williams F, Crichton M, et al. Effectiveness and implementation of models of cancer survivorship care: an overview of systematic reviews. *J Cancer Surviv*. Feb 2023;17(1):197-221. doi:10.1007/s11764-021-01128-1
7. Mollica MA, McWhirter G, Tonorezos E, et al. Developing national cancer survivorship standards to inform quality of care in the United States using a consensus approach. *J Cancer Surviv*. Aug 2024;18(4):1190-1199. doi:10.1007/s11764-024-01602-6
8. Smith AW, Parsons HM, Kent EE, et al. Unmet Support Service Needs and Health-Related Quality of Life among Adolescents and Young Adults with Cancer: The AYA HOPE Study. *Front Oncol*. 2013;3:75. doi:10.3389/fonc.2013.00075
9. Smith AW, Keegan T, Hamilton A, et al. Understanding care and outcomes in adolescents and young adult with Cancer: A review of the AYA HOPE study. *Pediatr Blood Cancer*. Jan 2019;66(1):e27486. doi:10.1002/pbc.27486
10. Zebrack B, Isaacson S. Psychosocial care of adolescent and young adult patients with cancer and survivors. *J Clin Oncol*. Apr 10 2012;30(11):1221-6. doi:10.1200/JCO.2011.39.5467
11. Jones JM, Fitch M, Bongard J, et al. The Needs and Experiences of Post-Treatment Adolescent and Young Adult Cancer Survivors. *J Clin Med*. May 13 2020;9(5)doi:10.3390/jcm9051444
12. Walsh C, Currin-McCulloch J, Simon P, Zebrack B, Jones B. Shifting Needs and Preferences: Supporting Young Adult Cancer Patients During the Transition from Active Treatment to Survivorship Care. *J Adolesc Young Adult Oncol*. Apr 2019;8(2):114-121. doi:10.1089/jayao.2018.0083
13. Crowder SL, Sauls R, Gudenkauf LM, et al. The Lived Experience of Young Adult Cancer Survivors after Treatment: A Qualitative Study. *Nutrients*. Jul 14 2023;15(14)doi:10.3390/nu15143145
14. Hydeman JA, Uwazurike OC, Adeyemi EI, Beaupin LK. Survivorship needs of adolescent and young adult cancer survivors: a concept mapping analysis. *J Cancer Surviv*. Feb 2019;13(1):34-42. doi:10.1007/s11764-018-0725-5
15. Arem H, Duarte DA, White B, et al. Young Adult Cancer Survivors' Perspectives on Cancer's Impact on Different Life Areas Post-Treatment: A Qualitative Study. *J Adolesc Young Adult Oncol*. Oct 2024;13(5):748-759. doi:10.1089/jayao.2024.0021
16. Parsons HM, Harlan LC, Schmidt S, et al. Who Treats Adolescents and Young Adults with Cancer? A Report from the AYA HOPE Study. *J Adolesc Young Adult Oncol*. Sep 2015;4(3):141-50. doi:10.1089/jayao.2014.0041
17. Fitch MI, Nicoll I, Lockwood G, Chan RJ, Grundy P. Adolescent and Young Adult Perspectives on Challenges and Improvements to Cancer Survivorship Care: How Are We Doing? *J Adolesc Young Adult Oncol*. Aug 2021;10(4):432-442. doi:10.1089/jayao.2020.0097
18. Arnett JJ. Emerging adulthood - A theory of development from the late teens through the twenties. *American Psychologist*. May 2000;55(5):469-480. doi:Doi 10.1037//0003-066x.55.5.469
19. Network NCC. NCCN clinical practice guidelines in oncology: Adolescent and young adult (AYA) oncology.: National Comprehensive Care Network; 2019.
20. Sahler O, Fairclough D, Phipps S, et al. Using problem-solving skills training to reduce negative affectivity in mothers of children with newly diagnosed cancer: Report of a multisite randomized trial. *Journal of Consulting and Clinical Psychology*. 2005;73(2):272-283.

21. Sahler O, Varni J, Fairclough D, et al. Problem-solving skills training for mothers of children with newly diagnosed cancer: A randomized trial. *Journal of Developmental & Behavioral Pediatrics*. 2002;23(2):77-86.
22. Sahler OJZ, Dolgin MJ, Phipps S, et al. Specificity of problem-solving skills training in mothers of children newly diagnosed with cancer: Results of a multisite randomized clinical trial. *Journal of Clinical Oncology*. 2013;31(10):1329-1335.
23. Lee AC, Driban JB, Price LL, Harvey WF, Rodday AM, Wang C. Responsiveness and minimally important differences for 4 patient-reported outcomes measurement information system short forms: physical function, pain interference, depression, and anxiety in knee osteoarthritis. *The Journal of Pain*. 2017;18(9):1096-1110.
24. Hennink M, Kaiser BN. Sample sizes for saturation in qualitative research: A systematic review of empirical tests. *Soc Sci Med*. Jan 2022;292:114523. doi:10.1016/j.socscimed.2021.114523
25. Hertzog MA. Considerations in determining sample size for pilot studies. *Res Nurs Health*. Apr 2008;31(2):180-91. doi:10.1002/nur.20247
26. D'Zurilla T, Nezu A, Maydeu-Olivares A. Social problem-solving inventory-revised (SPSI-R): Technical manual. *North Tonawanda, NY: Multi-Health Systems*. 2002;
27. Schepers S, Phipps S, Devine KA, et al. Psychometric properties of the 52-, 25-, and 10-Item Social Problem-Solving Inventory-Revised. under review;
28. Pilkonis PA, Choi SW, Reise SP, Stover AM, Riley WT, Cella D. Item banks for measuring emotional distress from the Patient-Reported Outcomes Measurement Information System (PROMIS®): depression, anxiety, and anger. *Assessment*. 2011;18(3):263-283.
29. Bevans M, Ross A, Cella D. Patient-Reported Outcomes Measurement Information System (PROMIS): Efficient, standardized tools to measure self-reported health and quality of life. *Nursing Outlook*. 2014;62(5):339-345.
30. Cella D, Riley W, Stone A, et al. Initial Adult Health Item Banks and First Wave Testing of the Patient-Reported Outcomes Measurement Information System (PROMIS(™)) Network: 2005–2008. *Journal of clinical epidemiology*. 08/04 2010;63(11):1179-1194. doi:10.1016/j.jclinepi.2010.04.011
31. Schalet BD, Pilkonis PA, Yu L, et al. Clinical validity of PROMIS depression, anxiety, and anger across diverse clinical samples. *Journal of clinical epidemiology*. 2016;73:119-127.
32. Pilkonis PA, Yu L, Dodds NE, Johnston KL, Maihoefer CC, Lawrence SM. Validation of the depression item bank from the Patient-Reported Outcomes Measurement Information System (PROMIS®) in a three-month observational study. *Journal of psychiatric research*. 2014;56:112-119.
33. Brucker PS. General Population and Cancer Patient Norms for the Functional Assessment of Cancer Therapy-General (FACT-G). 2005;28(2):192-211. doi:10.1177/0163278705275341
34. De Souza JA, Yap BJ, Hlubocky FJ, et al. The development of a financial toxicity patient-reported outcome in cancer: The COST measure. *Cancer*. 2014;120(20):3245-3253. doi:10.1002/cncr.28814
35. Palmer S, Patterson P, Thompson K. A national approach to improving adolescent and young adult (AYA) oncology psychosocial care: The development of AYA-specific psychosocial assessment and care tools. 2014;12(03):183-188. doi:10.1017/s1478951512001083
36. Kwak M, Zebrack BJ, Meeske KA, et al. Trajectories of Psychological Distress in Adolescent and Young Adult Patients With Cancer: A 1-Year Longitudinal Study. *Journal of Clinical Oncology*. May 6, 2013 2013;doi:10.1200/jco.2012.45.9222
37. Sidani S, Epstein DR, Fox M. Psychometric evaluation of a multi-dimensional measure of satisfaction with behavioral interventions. *Research in nursing & health*. 2017;40(5):459-469. doi:10.1002/nur.21808

38. Stoddard J, Reynolds E, Paris R, et al. The Coronavirus Impact Scale: Construction, Validation, and Comparisons in Diverse Clinical Samples. *JAACAP Open*. Jun 2023;1(1):48-59. doi:10.1016/j.jaacop.2023.03.003
39. Waheed W, Hughes-Morley A, Woodham A, Allen G, Bower P. Overcoming barriers to recruiting ethnic minorities to mental health research: a typology of recruitment strategies. *BMC Psychiatry*. 2015;15(1)doi:10.1186/s12888-015-0484-z
40. Jennrich RI, Schluchter MD. Unbalanced repeated-measures models with structured covariance matrices. *Biometrics*. 1986:805-820.
41. Thabane L, Mbuagbaw L, Zhang S, et al. A tutorial on sensitivity analyses in clinical trials: the what, why, when and how. *BMC Med Res Methodol*. Jul 16 2013;13:92. doi:10.1186/1471-2288-13-92
42. Preacher KJ, Hayes AF. Asymptotic and resampling strategies for assessing and comparing indirect effects in multiple mediator models. *Behav Res Methods*. Aug 2008;40(3):879-91. doi:10.3758/brm.40.3.879

RUTGERS
THE STATE UNIVERSITY OF NEW JERSEY

TRACK CHANGES LOG:

MOD #	DATE APPROVED	SUMMARY OF CHANGES	DOCUMENTS AFFECTED AND APPROVED VERSIONS
1	6/23/2020	1. MSK: Determination letter for reliance agreement uploaded.<br>2. Protocol: minor change.<br>In the Research Procedures section (pg.3). Participant contact information now will be collected immediately after consenting and prior to baseline survey (change from collecting in the baseline survey)<br>3. In addition, we expect most of the patient facing activities will now be virtual. Proper wording has been added in the Protocol to reflect these options.<br>4. We are adding recruitment materials: cover letter and info sheet, recruitment email and letter, recruitment script, recruitment flyer.<br><br>IRB requested additional changes:<br>Re: IRB response to minor changes to recruitment materials<br>1. All recruitment materials are now on Rutgers letterhead.<br>2. The PI is now the primary contact on recruitment materials.<br>3. Details about how and when we will use the recruitment materials are now included in the protocol under Recruitment details in section 4.1 (Subject Selection and Enrollment Considerations).	• Protocol: version 6.19.20<br>• Recruitment documents: (new)<br>• Recruitment E-mailL_V1_6.19.20<br>• Recruitment Letter_V1_6.19.20<br>• recruitment script_v1_6.19.20<br>• Study Information sheet_v1.1_6.19.20<br>• YA_PSST_Flyer_BrightIdeas.V1.1_6.19.20<br>• CoverLetterandInformationSheet_v1.1_6.19.20
2	9/15/2020	Requested: (9/14/2020)<br>1. Study Sites: To address prior stipulations, in eIRB section 5.1 we are adding the site information for Moffitt Cancer Center along with the executed reliance agreement for this site. Three forms are uploaded: HRP-1812B, SMART reliance agreement, and Moffitt's Informed Consent policy. Moffitt does not have its own IRB and is submitting their consent form for Rutgers IRB approval. This consent follows Rutgers' template but has the contact information for the Site PI at Moffitt. Please note the draft consent in HRP-1812B matched the language in the currently approved Rutgers consent; the uploaded document reflects the changes outlined in this modification so it is consistent with the Rutgers consent. This consent is uploaded in eIRB section 13.17.<br>2. Study Sites: To address prior stipulations, we are uploading the MSK site addendum, along with the provisional approval from their IRB/privacy Board, as well as IRB reliance determination letter.<br>3. Protocol changes: We made several minor changes to the protocol to enhance clarity, particularly around the options for remote procedures. We also propose increasing the sample size to account for higher drop-out rates than initially proposed and made some changes to the study surveys. Tracked and clean copies uploaded in eIRB application section 7.0. All changes are specified here:	• Protocol: version 8.20.2020<br>• Consent: version 8.20.2020<br>• BrightIDEAS-Survey1paper_v1.9.8.2020<br>• BrightIDEAS-Survey2paper_v1.9.8.2020<br>• BrightIDEAS-Survey3paper_v1.9.8.2020<br>• BrightIDEAS-Survey4paper_v1.9.8.2020<br>• CoverLetterandInformationSheet_v2_8.26.20_<br>• Study Information sheet_v2_8.26.20_<br>• Eligibility checklist.V2_2020-8-27_<br>• recruitment script_v8.26.20_CLEAN<br>• Script to obtaining consent_v1 8.31.20<br>• MedicalRecordAbstractionForm_9.8.2020<br><br>MSK site (uploaded documents):<br>MSK_PSY20-008_ExpeditedIRBApprovedPendingExternalIRBApproval149257~1

		- On p.1, we updated the PI's Division and Department to reflect recent change to Pediatrics/Division of Pediatric Hematology/Oncology.<br>- We are adding a table at the end of the Protocol document to document all modifications to the protocol.<br>- Section 1.1 Purpose/Specific Aims. We made minor wording change to clarify that the purpose is to evaluate efficacy. We are increasing the total sample size of young adult patients from 300 to 344 to allow for a 30% drop-out rate (we previously estimated a 20% drop-out rate based on our pilot study, but the main outcome is at a longer time point than the pilot so we now estimate greater drop-out). We also updated the sample size in eIRB application section 7.1/1.0 Study Overview/summary of the project.<br>- Section 1.3 A. Research Procedures. The randomization scheme stratified by age will now be for age groups 18-29 and 30-39. We have removed clinician rating of treatment intensity. We clarified many procedures that will now be completed online vs in-person. Subjects will now be notified of their random assignment via an automatic email via DatStat. We also specified that study staff will mail study subjects hard copies of materials (i.e., the Bright IDEAS manual, worksheets, and NCCN resources-depending on random assignment).<br>- Section 1.5 Sample Size Justification: We have changed the total study sample to 344 to accommodate a 30% dropout rate instead of 20%. (see also changes in section 4.3 Number of Subjects, A & B).<br>- Section 1.6 Study Variables: we specified that video-conference platform to be used in the study will be HIPAA compliant.<br>- Section 1.9 Data Collection. Location. We clarified the language regarding survey completion at home.<br>- Section 1.9 Data Collection. Procedures and Audio/Visual Recording. We will now use the Rutgers BOX, a cloud-based file storage application for secure storage, management and sharing of study digital files. We specified that only authorized members of the research team will be given access to this storage. This eliminates previously used LiFT Rutgers secure file transfer service.<br>- Section 1.9 Data Collection. Study Instruments. We made some clarifications to the descriptions of study instruments and their administration. We changed the name of "Psychosocial Care Received" to "Supportive Services Received" to better reflect the range of services covered. We added two new measures – PROMIS Social Isolation-Short Form 4a and Coronavirus Impact Scale to captures these outcomes.<br>- Section 1.9 Data Collection. Subject Identifiers: we added MRN, which will be used to abstract medical treatment data, and clarified that we will remove these identifiers from the research database at the conclusion of the study.<br>- Section 1.9 B. We clarified what data will be abstracted from the medical record and have uploaded the Data Abstraction Form.<br>- Section 3.0 Multi-center research: Clarified that trainings can be done remotely.	MSK_PSY20-008_MSK Addendum<br>Devine_3023_Determination_Letter (reliance determination letter)<br><br>MCC site (uploaded documents):<br>MCCMRI-P.CRO.02 - Informed Consent<br>MCC20551.RU HRP-1812B - FORM - Non Clinical Site 10.3.2019<br>Devine_3023_site_specific_determination_letter_Moffitt Cancer Center<br><br>*MCC consent submitted:*<br>MCC Consent20551.icf.v1.2020-08-20

		- Section 4.1A. Method to Identify Potential Subjects: Clarified that treating physicians or other qualified treatment team member familiar with the patient can assess medical eligibility.<br>- Section 4.1C. Inclusion/exclusion – we made minor clarifications to the language for the criteria. All criteria are the same but we separated diagnosis and treatment into two separate criteria and moved the exclusion regarding surgery-only to the Exclusion criteria.<br>- Section 4.3A/B: Updated total number of subjects from 300 to 344.<br>- Section 4.4. Consent Procedures. C. Documentation of Consent. We will consent subjects online via DatStat online consenting platform with electronic signature. The consent will now include a few questions to check understanding prior to signing.<br>- Section 4.6 C: We clarified we will keep records of compensation in DatStat.<br>- Section 4.7 Risk of Harm/Potential for Benefits to Subjects. D. Assessment of Social Behavior Consideration: We will monitor for participant distress by reviewing their distress scores on the Adolescent and Young Adult Oncology Screening Tool (scale 0-1) instead of the PROMIS measure. This will allow for more rapid review of the score and implementation of safety measures as needed. We also made this change in Section 6.3A – Data/Safety Monitoring Plan.<br>- Section 4.7 E Provisions to protect privacy: Clarified that any member of the treatment team can indicate if the patient is not suitable to approach.<br>4. Eligibility checklist: Changes made were to improve the clarity of the eligibility criteria and process. This will be programed in DatStat. Tracked and clean versions were uploaded in eIRB application section 7.0.<br>5. Study info change: number of subjects/sample size: from “Three hundred” to “Three hundred forty-four”. (Section 7.1 Study overview)<br>6. Recruitment materials: Recruitment script, Cover letter and Information sheet were updated with the new sample size. Clean and tracked copies uploaded in eIRB application section 11.0.<br>7. Study Surveys: We are uploading study surveys 1-4 in eIRB section 7.0. These will be programmed in DatStat for secure online data capture.<br>8. Consent: We made the following changes to the consent to reflect the changes in this modification: -Total number of people expected to take part in the study updated (from 300 to 344). -we specified that the video-conference platform they will use in the study will be HIPAA compliant. In addition we specified that the audio recordings will be stored on a secure could-based file storing application vs password-protected computer. For clarity, we also erased a sentence about the Doxy.me, since it was out of place and confusing. PI’s division and department has been updated to reflect the recent change (specified in Protocol changes above). In the section “Who may use, share or receive my information?” we now list the two sites for Non-Rutgers investigators on the team (removed the individual names for ease of understanding) and added the National Cancer Institute as the sponsor of this study, which was previously omitted by error. We also corrected a few typos. We added	

		a few questions to check understanding to help ensure that participants understand the study prior to signing the consent form. Finally, we moved the audio recording consent within the body of the main consent instead of having it as a consent Addendum, as this format will be easier to understand in the electronic format. Tracked and cleaned copies are uploaded in eIRB section 13.17.<br><br>The proposed changes are all minor and do not alter the risk/benefit ratio of the study.	
3	10/29/2020	(Requested 10/26/20)<br>We would like to make the following changes:<br>1. Personnel changes: Add Rutgers co-Investigators, Drs. Manne and Ohman Strickland. Add other study staff: Gary Kwok, Melissa Fleuhr, and Gabriella John, who will serve as interventionists; and Bachen xu as Data Analyst.<br>2. We are adding University of Rochester as an additional site "not engaged" in research. This site will contribute intellectually to the research and access coded audio recordings to conduct treatment fidelity ratings, but will not store any data or have access to any PHI or the code linking data and subject identifiers. A letter of cooperation is uploaded.<br>3. In section 5.1 we are uploading the MCC's Scientific Review Committee (SRC) and MSK's Privacy Board Committee's approvals of study changes to-date.<br>4. In section 7.1 Study Overview: in Study description we added 24 months follow-up assessment.<br>5. We have made minor changes to Time 2 Survey: we added one question about program feedback and made minor formatting and response scale correction (p. 10). Clean and tracked versions of T2 survey are uploaded.<br>6. We are adding a longer follow-up survey at 24 months after baseline. Time 5 Survey is uploaded.<br>Participants will receive $25 for completing this additional survey. We will also give a $25 bonus if they complete all 5 surveys for a total amount up to $150. (all study recruitment and consent documents affected have been changed to reflect the new time point for the additional survey (longer study duration per subject), an additional $25 gift card payment, $25 bonus gift card, and the total amount.<br>7. We are uploading two new Letters to Participants to mail study materials after randomization, including a small gift (e.g., branded stress ball) as a token of appreciation for their time and effort.<br>8. We are also uploading the automated emails and text messages that participants will be sent, including the welcome email with randomization assignment, invitations and reminders to complete surveys, and thank you emails with gift card payments.<br>9. We have updated the protocol to reflect the above changes. Specifically:<br>a. Section 1.1 A on pg. 2: added 24-month time point.<br>b. Section 1.3 on pg. 3: added 24-month time point, updated duration, and updated payments.	• Protocol v4 10.06.20<br>• Consent 2020-10-6 (Rutgers)<br>• Consent 2020-10-6 (MCC)<br>• BrightIDEAS-Survey2paper_v2.10.06.2020<br>• BrightIDEAS-Survey5paper_v1.10.6.2020New<br>• CoverLetterand InformationSheet_v3_10.06.20<br>• Study Information sheet_v3_10.06.20<br>• Recruitment E-mail_v2_10.06.20<br>• Recruitment script_v10.06.20<br>• Script to obtain consent_v2 10.06.20<br>• LetterStudyMaterialsGIfts_10.07.20New<br>• Flyer_BrightIdeas.V2_10.06.20<br>• DatStat Msg to Participants_V1.10.06.20new<br>• LetterStudyGIft_10.07.20new<br>• LetterStudyMaterialsGifts_10.07.20new

RUTGERS
THE STATE UNIVERSITY OF NEW JERSEY

		c. Section 1.9 on pg. 6, 7 and 8: added 24-month time point.<br>d. Section 1.10 on pg. 9. Added 24-month time point in the Timetable.<br>e. Section 2.4 added University of Rochester.<br>f. Section 4.6 pg. 13 updated the Compensation/Incentives.<br>g. Section 6.1 pg. 15 updated with 24-month time point.	
**4**	**01/25/21**	**(Requested date: 1/11/2021)**	
		1. Please remove Celeste Jackson from study staff. Please add Doris Fonseca and Karen Jackson as regulatory staff and Ivelisse Mandato, who will serve as a trainer and research assistant on study.<br>2. Protocol changes:<br><br>We made minor changes to protocol to indicate where we will store study data that may contain PHI. Previously we specified the BOX will be used to store these data. We now will store these data either on one drive a secure cloud storage solution, or on the Dr. Devine's secure J drive (per IT recommendation). We will use RBHS LiFT a large file secure transfer service for audio-recording file transfers. Changes made on pg. 6, of the protocol.<br><br>3. We corrected a typo on pg. 9 of the protocol.<br>4. We modified the wording of one exclusion criterion for clarity on p. 11.<br><br>These changes are minor and do not alter the benefit/risk ratio.	Protocol v5 2020.12.16
**5**	**2/24/21**	Requested: 2/15/21	
		1. Minor wording changes to the communication sent to participants via the DatStat platform. We specified the type of electronic gift card (i.e., Amazon) they will be receiving and the platform where they can redeem it (i.e., Redeem at Amazon.com). Tracked and clean files uploaded.<br>2. Minor wording change in the Eligibility Checklist to clarify the exclusion criterion. Protocol change (pg. 11) has been made/approved in prior modification (Mod4) and we are making this minor wording change to the Eligibility Checklist to match the protocol.<br>3. Minor wording changes to clarify instructions to online surveys are made to all 5 surveys. In addition, we have added a new question to the baseline (T1) questionnaire to gather data on how likely they are to get a COVID-19 vaccine once it is available for them. Tracked and clean copies of all questionnaires are uploaded.<br>These changes are all minor and do not alter the risk/benefit ratio of the study.	BrightIDEAS-Survey1paper_v2.1.27.20221CLEAN<br>BrightIDEAS-Survey2paper_v3.1.27.20221CLEAN<br>BrightIDEAS-Survey3paper_v2.1.27.20221CLEAN<br>BrightIDEAS-Survey4paper_v2.1.27.20221CLEAN<br>BrightIDEAS-Survey5paper_v2.1.27.20221CLEAN<br>DatStat Msg to Participants_V2.2.15.2021CLEAN<br>Eligibility checklist.V2_2021-2-15_CLEAN
**6**	**11/16/21**	Requested: 10/21/2021 (with 2021 CR)<br>We are requesting the following changes to the study:<br>- Please remove Gabriella John, Melissa Fleuhr and Baichen Xu from study staff as they are no longer on the project/at Rutgers Cancer Institute of New Jersey.	Protocol v6 2021-10-20<br>MedicalRecordAbstractionForm_10.20.2021<br>Eligibility checklist.V4_2021-10-19

		- Protocol pg. 8, minor change to clarify that any qualified member of the research team is now able to review audio recordings for treatment integrity regardless of site. It is not practical or necessary to assign treatment integrity review to someone from a different site, as the small team of reviewers are trained to complete the ratings without bias regardless of site.<br>- Protocol pg. 9, Secondary data collection. We have previously specified that we will access medical records of subjects to abstract medical data at the time of last survey follow-up. We are now clarifying that for subjects who do not remain in the study till last follow-up due to various reasons (i.e., no longer interested or subject deceased), we will access their medical records for data abstraction at time we exit them from the study, or as soon as practical.<br>- Protocol pg. 11 Inclusion Criteria. We have not changed the criteria however we further clarified that (1) patients who were previously diagnosed with non-melanoma skin cancers, treated with surgery only, can participate. (2) For types of treatment, we added in parentheses immunotherapy as an acceptable form of treatment for the criteria.<br>- Protocol pg. 13 Section 4.6 B Compensations/Incentives. We added a sentence about mailing Holiday cards to subjects on study in order to promote retention.<br>- Medical record abstraction form. Minor modification to add some additional fields, such as Other conditions documented (i.e., secondary malignancy, chronic disease(s), or mental health), first and last treatment dates, and deceased date (if applicable). Tracked and clean copies uploaded.<br>These changes are minor and do not alter the benefit/risk ratio.	
**7**	**11/23/21**	Please add Madeline Bono to study staff who will serve as a trainer.	None
**8**	**2/3/22**	Add Lohit Sodagum and Shengguo Li (study statistician) to study staff.	None
**9**	**5/23/22**	Add Molly Stern to study staff who will serve as a trainer.	None
**10**	**8/26/22**	We would like to make the following minor changes to the study.<br>1. Participant incentives: Currently, subjects receive a $25 gift card for each completed survey (total of 5) and an additional $25 gift card bonus if they complete all surveys (total of up to $150). Given participants' time/effort to complete the last follow-up at two years post-enrollment, we would like to provide $50 for their effort at that time instead of offering $25 plus the bonus. The total compensation does not change. We also feel this is more fair to all participants and will yield better retention of subjects in the study. This requires modification to all documents that list the incentive: protocol (pg. 13, track changes log on pg. 20), informed consent form, as well as all recruitment documents, i.e., Cover Letter and Information Sheet, Recruitment e-mail, Recruitment script, Study Information sheet, and Study flyer. For participants already enrolled on the study, we will	Protocol_BrightIDEASYAv7_08.08.2022<br>Communications_new_mod10_v1_8.8.2022<br>Recruitment E-mailL_V2_08.08.22<br>recruitment script_v8.08.22<br>MedicalRecordAbstractionForm_8.8.22<br>DatSat msg to Participants_V3_08.08.2022<br>Consent 2022_08_08<br>MCC Consent 2022-08-08<br>CoverLetterand InformationSheetv4_08.08.2022<br>Study Information sheet_v4_08.08.22<br>YA_PSST_Flyer_BrightIdeas_8.8.22

		notify them of the change via email that will contain a link to download the revised version of the consent form for their records (see Communications_new_mod10_v1_8.8.22.doc).<br>2. We have added a new communication to study participants to promote retention: There are 12 months between Survey 4 and Survey 5 when no other study activities occur. We would like to contact the subjects via email at about 18 months since study enrollment to (1) Thank them for continued participation in our study, and (2) ask them to notify us of any contact information changes (mailing address, phone numbers, etc). See Communications_new_mod10_v1_8.8.22.doc<br>3. Medical Record Abstraction form has been modified. We simplified the disease and treatment information to be collected, specified the types of documented psychosocial conditions and social concerns, specified the types of psychosocial services used, and added documented hospice referral and Covid-19 vaccination status (initial and booster). Tracked and clean versions uploaded: MedicalRecordAbstractionForm_8.8.22.doc. Protocol was also modified on pg. 9.<br>4. Staff changes: we have removed Doris Fonseca (no longer with OHRS) and added Kassie DiOrio as regulatory staff. We removed Madelyn Bono who is no longer working on the study.<br>These changes are minor and do not alter the benefit/risk ratio.	
**11**	**10/13/22**	Staff change: Please add Christian Bean to the study staff who will serve as a Trainer to deliver the intervention to the subjects.	
**12**	**1/11/23**	We are requesting the following changes:<br>1. Staff changes: Add Madeline Bono to study staff.<br>2. We would like to increase the number of subjects currently approved in section 10.0 by 20%, (from n=344 to n= 413), to account for patients who sign the consent, but never complete any study-related tasks and therefore are removed from the study due to "non-compliance" or "other" reasons. Our study goal sample remains unchanged. We expect a total sample of n=344 subjects to enroll (i.e., consent + complete baseline + get randomized into one of the study arms.)<br>3. We would like to make changes to the final survey (Survey 5):<br>a. We are adding additional measures on Covid-19 vaccine/booster shot and attitudes, and Cancer Care Follow-up and survivorship.<br>b. At the end of the survey we are requesting consent to contact the subjects about participation in future studies, for example, if we were to do a follow-up study in the future. (yes/no). This will allow us to store their contact information (i.e., first and last names, phone numbers, email and mailing addresses) as well as relevant cancer history (i.e., diagnosis, date of diagnosis, and end of treatment date) on the PI's secure OneDrive for up to 10 years, with access granted only to the PI, study coordinator, and limited authorized research staff. We notify the subjects that the participation in the future studies is voluntary and they are at no obligation to participate in the future. Changes made to Protocol on pg. 16 to specify these data storage and security.	Protocol_BrightIDEASYAv8_12.20.2022<br>BrightIDEAS-Survey5paper_v3.12.20.2022

		(see tracked and clean copies of the Survey 5)<br><br>These changes are minor and do not alter the benefit/risk ratio.	
**13**	**4/7/2023**	We are requesting the following changes:<br>1. Please add Harrison Clement (statistician/research assistant) and Hanna Brancaccio (research assistant) to study staff.<br>2. We would like to make a slight change to the Comprehensive Score for Financial Toxicity (COST) measure administered at 12 months and 24 months. Presently, the full 11-item COST measure is administered to those, who indicate a primary responsibility paying for their medical care. Those who indicate they are not primarily responsible, are only administered the 2 out of 11 items of the measure. After reviewing the data to-date, we would like to make the change to 12 month and 24 month surveys, so that the full 11-item COST measure is administered to the whole sample, regardless of their level of financial independence/responsibility. Tracked and clean versions of Protocol, Survey 4, and Survey 5 are attached.<br>These changes are minor and do not alter the risk/benefit ratio of the study.	Protocol_BrightIDEASYAv9_3.15.2023<br>BrightIDEAS-Survey4paper_v3.3.15.2023<br>BrightIDEAS-Survey5paper_v4.3.15.2023
**14**	**7/19/2023**	Site PI change at the Moffitt Cancer Center: Dr. Donovan, current PI for MCC, will be leaving the institution (last day 7/14/2023). Dr. Lora M. Thompson will be the new PI for the Moffitt Cancer Center site on this study. This requires an update to HRP-1812B form (uploaded to NSF 5.1) and local site consent form (uploaded to NSF 5.1 & section 13.17.	MCC Consent20551.icf.v4.2023-07-10
**15**	**7/24/2023**	Please remove Ivelisse Mandato, Gary Kwok, and Lohit Sodagum from study staff. Please add Meredith Collins, Angela Senger, and Melanie Spruill.	NA
**16**	**10/20/2023**	Please add Elizabeth Glaser to study personnel and removed Christian Bean and Hanna Brancaccio.	NA
**17**	**9/20/2024**	Please remove Elizabeth Glaser, Melanie Spruill and Harrison Clement, and add Angelina Resal, and Raynold Pandi to the study.	NA
**18**		1. We were awarded an administrative supplement to conduct qualitative interviews with a subset of 30 young adults from the parent award randomized controlled trial. We will approach and consent 30 young adults from the parent trial who agreed for future contact. Details are added throughout the protocol. Recruitment script, consent form, and the interview guide are uploaded.<br><br>2. We were also awarded a "merit extension" supplement to extend the grant 2 additional years and carry out related aims in that period. This work will identify barriers and	Protocol_BrightIDEASYAv10_2.18.2025<br>Extension Supplement_KeyInformant InterviewConsent_v1_2.18.2025<br>Extension Supplement_Feasibility pilot_Recruitment Email_Script v1_2.26.2025<br>Extension Supplement_Feasibility pilot Consent 2.18.2025<br>Extension Supplement_AdaptationFGConsent_v1_2.18.2025<br>Eligibility checklist Feasibility pilot v1_2.26.2025<br>Eligibility checklist Adaptation_v1_2.26.2025

		facilitators of disseminating Bright IDEAS-YA to other settings (through review of existing audio recordings and new qualitative interviews) and address one barrier to access by culturally and linguistically tailoring to young adults who identify as Hispanic and/or are Spanish-speaking. This work will involve up to 70 additional participants (up to 50 for qualitative work, 20 for a feasibility pilot of the culturally adapted Bright IDEAS-YA). Details are added throughout the protocol. Consent forms for focus group, key informant interviews, and the feasibility pilot are uploaded. We will finalize, translate into Spanish, and obtain IRB approval for any additional documents required for the conduct of the protocol for the supplemental award.<br><br>In addition, we removed the name of the protocol template in the footer, updated a reference for Coronavirus Impact Scale on pg. 11, and updated the tracked changes log at the end.<br><br>Staff changes requested: Please add Denalee O'Malley and Evelyn Arana as co-investigators, and Jenna Howard, Arlette Chavez Iniguez, Rachel Anca, and Rinat Heller to study staff.<br><br>Section 1.3 was updated with Family Medicine and Community Health department of RWJMS (Dr. O'Malley)<br><br>Section 7.0: Study type has also check for "Pilot".<br><br>Section 7.1: Summary of the project was updated to reflect the addition of the supplemental work.<br><br>Section 10.0: Subject Population, "All Genders" is now also checked. (1.0) & total N subject has been increased to accommodate the supplemental work.	AdminSupplement_InterviewGuide_v1_2.26.25<br>AdminSupplement_Bright IDEAS YA_Recruitment Script_v1_2.20.2025<br>AdministrativeSupplement_InterviewConsent_v1_2.18.2025
