# Supplementary material for: Bright IDEAS-YA Skills Training and Psychosocial Outcomes of Young Adults With Cancer: A Randomized Clinical Trial
Source: JAMA Netw Open. 2026 Apr 21;9(4):e267997. doi: 10.1001/jamanetworkopen.2026.7997 (PMC13100872; doi:10.1001/jamanetworkopen.2026.7997)
Supplement: Supplement 2. — eAppendix. Supplementary Data [file jamanetwopen-e267997-s002.pdf]

Supplemental Online Content

Devine KA, Barnett M, Donovan KA, et al. Bright IDEAS-YA skills training and psychosocial outcomes of young adults with cancer. *JAMA Newt. Open*. 2026;9(4):e267997. doi:10.1001/jamanetworkopen.2026.7997

eAppendix. Supplementary Data

This supplemental material has been provided by the authors to give readers additional information about their work.

© 2026 Devine KA et al. *JAMA Network Open*.

eAppendix. Supplementary Data

Supplemental Table 1. Baseline characteristics for participants with and without missing outcome values at six-months (T3).

**Characteristic**	**T3 Missed** n = 64 n (%)	**T3 Completed** n = 280 n (%)	**Statistic (p-value)**[1]
**Age**			0.07 (0.90)
18-29	30 (46.88%)	126 (45.00%)	
30-39	34 (53.13%)	154 (55.00%)	
**Sex**			0.80 (0.42)
Male	27 (42.19%)	101 (36.20%)	
Female	37 (57.81%)	178 (63.80%)	
**Race**			4.89 (0.29)
White	46 (71.88%)	176 (62.86%)	
Black or African American	9 (14.06%)	29 (10.36%)	
Asian	4 (6.25%)	39 (13.93%)	
Other	3 (4.69%)	23 (8.21%)	
Unknown/missing	2 (3.13%)	13 (4.64%)	
**Hispanic Ethnicity**			0.001 (>0.99)
Yes	12 (18.75%)	52 (18.57%)	
No	52 (81.25%)	228 (81.43%)	
**Employment Status**			0.20 (0.90)
Student Part-Time/Full-Time	11 (17.19%)	43 (15.47%)	
Working Part-Time/Full-Time	47 (73.44%)	205 (73.74%)	
Unemployed/Homemaker/Caregiver	6 (9.38%)	30 (10.79%)	
Missing	0	2	
**Highest Grade Completed**			**6.75 (0.009)**
Less than HS/High School combined	26 (41.27%)	70 (25.00%)	
Some College/4-year/grad degree	37 (58.73%)	210 (75.00%)	
Missing	1	0	
**Health Insurance**			3.65 (0.30)
Private	50 (78.13%)	227 (81.36%)	
Public/Public and Private	13 (20.31%)	37 (13.26%)	
Military	1 (1.56%)	10 (3.58%)	
None	0 (0.00%)	5 (1.79%)	
Missing	0	1	

© 2026 Devine KA et al. *JAMA Network Open*.

**Characteristic**	**T3 Missed**<br>n = 64<br>n (%)	**T3 Completed**<br>n = 280<br>n (%)	**Statistic (p-value)[1]**
**Income**			1.67 (0.65)
<$50k	13 (20.63%)	49 (18.56%)	
$50k - <$100k	12 (19.05%)	61 (23.11%)	
>$100k	23 (36.51%)	107 (40.53%)	
Don't know/Prefer not to answer	15 (23.81%)	47 (17.80%)	
Missing	1	16	
**Marital Status**			0.07 (>0.99)
Single, never married	29 (45.31%)	126 (45.00%)	
Married/Remarried	26 (40.63%)	113 (40.36%)	
Unmarried but living with partner	6 (9.38%)	29 (10.36%)	
Separated or Divorced	3 (4.69%)	12 (4.29%)	
**Cancer Diagnosis**			**8.95 (0.009)**
Blood	23 (35.94%)	111 (39.64%)	
Breast	9 (14.06%)	79 (28.21%)	
Others[2]	32 (50.00%)	90 (32.14%)	
**Anxiety**, Mean (SD)	57.05 (9.09)	58.92 (7.65)	7,587 (0.06)
**Depression**, Mean (SD)	52.15 (9.74)	52.80 (8.64)	8,457 (0.54)
**HRQOL**, Mean (SD)	70.90 (17.18)	69.98 (15.32)	9,200 (0.64)

Note. [1]Exact Pearson's Chi-squared test (simulated p-value based on 2000 replicates) for categorical characteristics; Wilcoxon rank sum test for continuous characteristics

[2]Other diagnoses included sarcoma, colorectal, testicular, cervical/ovarian/endometrial, CNS tumor, gastric, head and neck, myeloma, neuroendocrine, thoracic/lung, and melanoma or other skin cancers.

© 2026 Devine KA et al. *JAMA Network Open*.

Supplemental Table 2. Analyses evaluating Baseline to Post-Intervention changes in Total SPSI-R:S score and subscales as mediators of treatment effects on depression, anxiety, and HRQOL.

	Depression		Anxiety		HRQOL	
	Estimate (95% CI)	*p*-value	Estimate (95% CI)	*p*-value	Estimate (95% CI)	*p*-value
**Total Problem-Solving Ability**						
Direct Effect	-3.30 (-5.15, -1.58)	<0.001	-2.31 (-4.03, -0.65)	0.006	3.66 (0.18, 7.09)	0.04
Indirect Effect	-0.36 (-0.83, -0.04)	0.08	-0.45 (-0.89, -0.09)	0.03	0.31 (-0.26, 1.11)	0.35
Total Effect	-3.66 (-5.47, -1.92)	<0.001	-2.75 (-4.40, -1.05)	0.001	3.97 (0.49, 7.26)	0.02
Proportion Mediated	0.10 (0.01, 0.26)	0.15	0.16 (0.03, 0.45)	0.20	0.08 (-0.12, 0.54)	0.87
**Positive Problem Orientation**						
Direct Effect	-3.59 (-5.27, -1.80)	<0.001	-2.63 (-4.31, -1.1)	<0.001	3.86 (0.61, 7.35)	0.03
Indirect Effect	-0.07 (-0.33, 0.14)	0.55	-0.12 (-0.41, 0.06)	0.24	0.12 (-0.21, 0.60)	0.54
Total Effect	-3.66 (-5.29, -1.87)	<0.001	-2.75 (-4.37, -1.21)	<0.001	3.97 (0.64, 7.37)	0.02
Proportion Mediated	0.02 (-0.04, 0.10)	0.55	0.04 (-0.02, 0.17)	0.24	0.03 (-0.07, 0.26)	0.55
**Negative Problem Orientation**						
Direct Effect	-2.93 (-4.62, -1.14)	<0.001	-2.29 (-3.86, -0.66)	0.001	3.64 (0.26, 7.16)	0.03
Indirect Effect	-0.73 (-1.36, -0.27)	<0.001	-0.46 (-0.95, -0.09)	0.01	0.33 (-0.34, 1.04)	0.32
Total Effect	-3.66 (-5.44, -1.87)	<0.001	-2.75 (-4.32, -1.07)	<0.001	3.97 (0.54, 7.46)	0.03
Proportion Mediated	0.20 (0.08, 0.44)	<0.001	0.17 (0.03, 0.46)	0.01	0.08 (-0.13, 0.56)	0.33
**Rational Problem Solving**						
Direct Effect	-3.55 (-5.28, -1.73)	<0.001	-2.61 (-4.17, -0.94)	0.002	3.74 (0.40, 7.28)	0.03
Indirect Effect	-0.06 (-0.32, 0.10)	0.45	-0.03 (-0.21, 0.17)	0.78	0.21 (-0.19, 0.80)	0.35
Total Effect	-3.62 (-5.32, -1.81)	<0.001	-2.63 (-4.22, -0.97)	<0.001	3.94 (0.73, 7.47)	0.02
Proportion Mediated	0.02 (-0.03, 0.10)	0.45	0.01 (-0.08, 0.11)	0.78	0.05 (-0.07, 0.39)	0.36
**Impulsivity/Carelessness Style**						
Direct Effect	-3.59 (-5.31, -1.83)	<0.001	-2.50 (-4.25, -0.82)	0.006	3.96 (0.81, 7.11)	0.02
Indirect Effect	-0.03 (-0.27, 0.10)	0.72	-0.13 (-0.51, 0.08)	0.27	-0.01 (-0.30, 0.24)	0.89
Total Effect	-3.62 (-5.34, -1.87)	<0.001	-2.63 (-4.40, -0.91)	0.002	3.94 (0.78, 6.99)	0.03
Proportion Mediated	0.01 (-0.03, 0.08)	0.72	0.05 (-0.04, 0.23)	0.27	-0.00 (-0.12, 0.10)	0.91
**Avoidant Style**						
Direct Effect	-3.55 (-5.32, -1.60)	<0.001	-2.47 (-4.00, -0.81)	0.006	3.90 (0.42, 7.22)	0.02
Indirect Effect	-0.06 (-0.32, 0.09)	0.51	-0.17 (-0.47, 0.10)	0.24	0.04 (-0.27, 0.46)	0.73
Total Effect	-3.62 (-5.36, -1.67)	<0.001	-2.63 (-4.17, -0.98)	0.002	3.94 (0.44, 7.26)	0.02
Proportion Mediated	0.02 (-0.03, 0.10)	0.51	0.06 (-0.05, 0.24)	0.24	0.01 (-0.15, 0.19)	0.73

Note. Mediation analyses examined the change in problem-solving ability from baseline to post-intervention (T1 to T2) as a predictor of the change in outcome variables from baseline to 6-months (T1 to T3). Depression is measured using PROMIS Depression T-Score, Anxiety is measured using PROMIS Anxiety T-Score, and HRQOL is measured using FACT-G Total Score. Problem-solving ability is measured using SPSI-R:S. CI = Confidence Interval

© 2026 Devine KA et al. *JAMA Network Open*.

Supplemental Table 3. Summary of moderation analysis for anxiety.

	Anxiety for Males		Anxiety for Females	
	**Estimate (95% CI)**	**p-value**	**Estimate (95% CI)**	**p-value**
**Baseline to 3-month Change**				
Control	0.15 (-1.58, 1.88)	0.86	-0.72 (-2.15, 0.71)	0.32
Intervention	-2.67 (-4.62, -0.72)	**0.007**	-2.04 (-3.45, -0.63)	**0.005**
Treatment Effect	-2.82 (-5.43, -0.22)	**0.03**	-1.32 (-3.33, 0.69)	**0.20**
**Baseline to 6-month Change**				
Control	1.57 (-0.21, 3.35)	0.08	-0.76 (-2.21, 0.69)	0.30
Intervention	-3.72 (-5.73, -1.71)	**<0.001**	-1.52 (-2.95, -0.08)	**0.04**
Treatment Effect	-5.29 (-7.97, -2.60)	**<0.001**	-0.75 (-2.79, 1.29)	0.47

Note. Statistically significant results are bolded. Analyses included all randomized participants (Intervention n = 171; Control n = 173). Anxiety is measured using PROMIS Anxiety T-Score. CI = Confidence Interval; Moderation as measured by three-way interaction between outcome, treatment, and time was significant, $F(2) = 7.08$, $p=0.03$.

© 2026 Devine KA et al. *JAMA Network Open*.

Supplemental Table 4. Moderation analyses

		Depression		Anxiety		HRQOL	
**Moderator**	**Term**	**F statistic (df)**	***p*-value**	**F statistic (df)**	***p*-value**	**F statistic (df)**	***p*-value**
**Sex**	Group	1.32 (1)	0.25	0.06 (1)	0.80	0.46 (1)	0.50
	Time	0.01 (2)	1.00	1.42 (2)	0.49	4.66 (2)	0.10
	Sex	1.77 (1)	0.18	8.10 (1)	0.004	1.59 (1)	0.21
	Group x Time	7.49 (2)	0.02	1.72 (2)	0.42	3.50 (2)	0.17
	Sex x Time	0.75 (2)	0.69	4.05 (2)	0.13	1.34 (2)	0.51
	Group x Sex	0.18 (1)	0.67	0.34 (1)	0.56	0.41 (1)	0.52
	Group x Time x Sex	0.48 (2)	0.79	7.08 (2)	0.03	1.27 (2)	0.53
**Race**	Group	0.53 (1)	0.47	0.47 (1)	0.49	0.53 (1)	0.47
	Time	0.80 (2)	0.67	0.63 (2)	0.73	5.07 (2)	0.08
	Race	5.64 (2)	0.06	0.88 (2)	0.64	1.17 (2)	0.56
	Group x Time	8.82 (2)	0.01	8.31 (2)	0.02	7.77 (2)	0.02
	Race x Time	12.31 (4)	0.02	0.42 (4)	0.98	1.69 (4)	0.79
	Group x Race	2.33 (2)	0.31	0.16 (2)	0.92	1.92 (2)	0.38
	Group x Time x Race	8.24 (4)	0.08	1.21 (4)	0.88	2.19 (4)	0.70
**Ethnicity**	Group	4.10 (1)	0.04	3.62 (1)	0.06	1.46 (1)	0.23
	Time	4.70 (2)	0.10	3.26 (2)	0.20	1.66 (2)	0.44
	Ethnicity	0.80 (1)	0.37	0.62 (1)	0.43	0.34 (1)	0.56
	Group x Time	9.38 (2)	0.009	12.03 (2)	0.002	2.52 (2)	0.28
	Ethnicity x Time	4.52 (2)	0.10	3.85 (2)	0.15	3.60 (2)	0.17
	Group x Ethnicity	1.90 (1)	0.17	2.88 (1)	0.09	0.45 (1)	0.50
	Group x Time x Ethnicity	3.66 (2)	0.16	5.57 (2)	0.06	0.23 (2)	0.89
**Financial Strain**	Group	1.10 (1)	0.29	0.13 (1)	0.72	0.62 (1)	0.43
	Time	3.32 (2)	0.19	0.76 (2)	0.59	4.37 (2)	0.11
	Financial Strain	6.97 (1)	0.008	4.60 (1)	0.03	7.52 (1)	0.006
	Group x Time	8.22 (2)	0.02	3.40 (2)	0.18	4.04 (2)	0.13
	Financial Strain x Time	5.66 (2)	0.06	0.07 (2)	0.96	0.001 (2)	0.99
	Group x Financial Strain	0.20 (1)	0.66	0.44 (1)	0.51	0.22 (1)	0.64
	Group x Time x Financial Strain	0.44 (2)	0.80	0.94 (2)	0.62	0.75 (2)	0.69
**Unmet Needs**	Group	2.19 (1)	0.14	0.10 (1)	0.76	0.11(1)	0.74
	Time	8.78 (2)	0.01	6.83 (2)	0.03	1.60 (2)	0.45
	Unmet Needs	57.89 (1)	<0.001	48.29 (1)	<0.001	43.13 (1)	<0.001
	Group x Time	10.47 (2)	0.005	5.80 (2)	0.06	1.68 (2)	0.43
	Unmet Needs x Time	15.53 (2)	<0.001	13.53 (2)	0.001	8.40 (2)	0.02
	Group x Unmet Needs	1.34 (1)	0.25	0.23 (1)	0.63	<0.001 (1)	0.98
	Group x Time x Unmet Needs	2.41 (2)	0.30	0.74 (2)	0.69	0.42 (2)	0.81

© 2026 Devine KA et al. *JAMA Network Open*.

**Distress**	Group	2.24 (1)	0.14	0.68 (1)	0.41	0.59 (1)	0.44
	Time	6.61 (2)	0.04	7.10 (2)	0.03	3.72 (2)	0.16
	Distress	35.45 (1)	<0.001	44.89 (1)	<0.001	28.10 (1)	<0.001
	Group x Time	7.86 (2)	0.02	11.18 (2)	0.004	3.58 (2)	0.17
	Distress x Time	11.65 (2)	0.003	15.93 (2)	<0.001	2.31 (2)	0.32
	Group x Distress	0.88 (1)	0.35	0.67 (1)	0.41	0.02 (1)	0.88
	Group x Time x Distress	2.69 (2)	0.26	3.17 (2)	0.21	0.08 (2)	0.96
**Education**	Group	0.98 (1)	0.32	0.66 (1)	0.42	0.32 (1)	0.57
	Time	2.03 (2)	0.13	1.95 (2)	0.14	0.51 (2)	0.60
	Education	0.04 (1)	0.85	0.31 (1)	0.58	0.38 (1)	0.54
	Group x Time	9.59 (2)	<0.001	9.07 (2)	<0.001	0.02 (2)	0.98
	Education x Time	1.98 (2)	0.14	2.20 (2)	0.11	0.05 (2)	0.96
	Group x Education	0.01 (1)	0.93	0.13 (1)	0.72	2.38 (1)	0.12
	Group x Time x Education	4.07 (2)	0.02	4.88 (2)	0.008	0.91 (2)	0.40

Note. Depression is measured using PROMIS Depression T-Score, Anxiety is measured using PROMIS Anxiety T-Score, and HRQOL is measured using FACT-G Total Score.

© 2026 Devine KA et al. *JAMA Network Open*.

Supplemental Table 5. Planned sensitivity analyses of linear models without site as a fixed factor on depression, anxiety, and HRQOL.

	**Depression**		**Anxiety**		**HRQOL**	
	**Estimate (95% CI)**	**p-value**	**Estimate (95% CI)**	**p-value**	**Estimate (95% CI)**	**p-value**
**Baseline to 3-month Change**						
Control	0.26 (-0.89, 1.42)	0.66	-0.40 (-1.50, 0.70)	0.48	-0.11 (-2.18, 1.97)	0.92
Intervention	-1.77 (-2.97, -0.57)	**0.004**	-2.25 (-3.39, -1.11)	**<0.001**	3.04 (0.88, 5.20)	**0.006**
Treatment Effect	-2.03 (-3.70, -0.36)	**0.02**	-1.85 (-3.44, -0.27)	**0.02**	3.15 (0.15, 6.14)	**0.04**
**Baseline to 6-month Change**						
Control	0.31 (-0.87, 1.49)	0.61	0.21 (-0.91, 1.33)	0.72	2.67 (0.56, 4.79)	**0.01**
Intervention	-2.93 (-4.15, -1.70)	**<0.001**	-2.24 (-3.40, -1.07)	**<0.001**	6.09 (3.88, 8.29)	**<0.001**
Treatment Effect	-3.24 (-4.94, -1.54)	**<0.001**	-2.44 (-4.06, -0.83)	**0.003**	3.41 (0.36, 6.47)	**0.03**

Note. Depression is measured using PROMIS Depression T-Score, Anxiety is measured using PROMIS Anxiety T-Score, and HRQOL is measured using FACT-G Total Score. CI = Confidence Interval

© 2026 Devine KA et al. *JAMA Network Open*.

Supplemental Table 6. Sensitivity analyses of the models including only intervention completers (*n* = 123) vs. EUC (*n* = 173).

	Depression		Anxiety		HRQOL	
	**Estimate (95% CI)**	**p-value**	**Estimate (95% CI)**	**p-value**	**Estimate (95% CI)**	**p-value**
**Baseline to 3-month Change**						
Control	0.26 (-0.87, 1.39)	0.65	-0.39 (-1.47, 0.69)	0.48	-0.11 (-2.13, 1.90)	0.91
Intervention	-1.64 (-2.95, -0.33)	**0.014**	-2.71 (-3.96, -1.46)	**<0.001**	4.45 (2.11, 6.79)	**<0.001**
Treatment Effect	-1.90 (-3.63, -0.17)	**0.031**	-2.32 (-3.97, -0.67)	**0.006**	4.56 (1.48, 7.65)	**0.004**
**Baseline to 6-month Change**						
Control	0.31 (-0.84, 1.47)	0.59	0.22 (-0.89, 1.32)	0.70	2.66 (0.61, 4.72)	**0.011**
Intervention	-3.01 (-4.35, -1.67)	**<0.001**	-2.52 (-3.79, -1.24)	**<0.001**	6.65 (4.26, 9.05)	**<0.001**
Treatment Effect	-3.32 (-5.09, -1.56)	**<0.001**	-2.73 (-4.42, -1.05)	**0.002**	3.99 (0.84, 7.14)	**0.01**

Note. Statistically significant results are bolded. Depression is measured using PROMIS Depression T-Score, Anxiety is measured using PROMIS Anxiety T-Score, and HRQOL is measured using FACT-G Total Score. CI = Confidence Interval.

Supplemental Table 7. Sensitivity analyses of linear models on depression, anxiety, and HRQOL adjusting for education (due to slight imbalance at baseline).

	Depression		Anxiety		HRQOL	
	**Estimate (95% CI)**	**p-value**	**Estimate (95% CI)**	**p-value**	**Estimate (95% CI)**	**p-value**
**Baseline to 3-month Change**						
Control	-0.27 (-0.89, 1.42)	0.65	-0.39 (-1.49, 0.71)	0.48	-0.12 (-2.19, 1.96)	0.91
Intervention	-1.75 (-2.95, -0.55)	**0.004**	-2.25 (-3.39, -1.11)	**<0.001**	3.02 (0.86, 5.18)	**0.006**
Treatment Effect	-2.02 (-3.68, -0.35)	**0.02**	-1.86 (-3.44, -0.27)	**0.02**	3.14 (0.14, 6.14)	**0.04**
**Baseline to 6-month Change**						
Control	0.33 (-0.85, 1.51)	0.58	0.22 (-0.90, 1.34)	0.72	2.64 (0.53, 4.76)	**0.01**
Intervention	-2.91 (-4.14, -1.69)	**<0.001**	-2.23 (-3.40, -1.06)	**<0.001**	6.06 (3.86, 8.27)	**<0.001**
Treatment Effect	-3.24 (-4.94, -1.54)	**<0.001**	-2.45 (-4.07, -0.83)	**0.003**	3.42 (0.36, 6.48)	**0.03**

Note. Depression is measured using PROMIS Depression T-Score, Anxiety is measured using PROMIS Anxiety T-Score, and HRQOL is measured using FACT-G Total Score. CI = Confidence Interval

© 2026 Devine KA et al. *JAMA Network Open*.

Supplemental Table 8. Sensitivity analysis stratifying by education.

	Depression				Anxiety			
	Lower Education		Higher Education		Lower Education		Higher Education	
	Estimate (95% CI)	p-value	Estimate (95% CI)	p-value	Estimate (95% CI)	p-value	Estimate (95% CI)	p-value
**Baseline to 3-month Change**								
Control	0.92 (-1.68, 3.52)	0.48	0.05 (-1.22, 1.32)	0.94	0.92 (-1.51, 3.35)	0.46	-0.82 (-2.03, 0.40)	0.19
Intervention	-3.54 (-5.90, -1.17)	**0.004**	-1.03 (-2.41, 0.36)	0.15	-3.49 (-5.73, -1.24)	**0.003**	-1.80 (-3.12, -0.48)	**0.008**
Treatment Effect	-4.46 (-7.97, -0.95)	**0.01**	-1.08 (-2.96, 0.80)	0.26	-4.41 (-7.71, -1.10)	**0.009**	-0.98 (-2.77, 0.81)	0.28
**Baseline to 6-month Change**								
Control	2.60 (-0.25, 5.45)	0.07	-0.27 (-1.54, 1.00)	0.68	2.37 (-0.29, 5.03)	0.08	-0.38 (-1.59, 0.84)	0.54
Intervention	-4.88 (-7.30, -2.45)	**<0.001**	-2.11 (-3.52, -0.70)	**0.003**	-4.57 (-6.88, -2.27)	**<0.001**	-1.29 (-2.63, 0.05)	0.06
Treatment Effect	-7.47 (-11.2, -3.74)	**<0.001**	-1.84 (-3.74, 0.05)	0.06	-6.94 (-10.5, -3.43)	**<0.001**	-0.92 (-2.73, 0.89)	0.32

Note. Statistically significant results are bolded. Depression is measured using PROMIS Depression T-Score, Anxiety is measured using PROMIS Anxiety T-Score, and HRQOL is measured using FACT-G Total Score. CI = Confidence Interval. Moderation as measured by three-way interaction between outcome, treatment, and time was significant for depression, F(2) = 4.07, p=0.02, and for anxiety, F(2) = 4.88, p=0.008.

© 2026 Devine KA et al. *JAMA Network Open*.
