# Supplementary material for: Bright IDEAS-YA Skills Training and Psychosocial Outcomes of Young Adults With Cancer: A Randomized Clinical Trial
Source: JAMA Netw Open. 2026 Apr 21;9(4):e267997. doi: 10.1001/jamanetworkopen.2026.7997 (PMC13100872; doi:10.1001/jamanetworkopen.2026.7997)
Supplement: Supplement 3. — Data Sharing Statement [file jamanetwopen-e267997-s003.pdf]

Data Sharing Statement

Devine. Bright IDEAS-YA Skills Training and Psychosocial Outcomes of Young Adults With Cancer. *JAMA Netw Open*. Published April 21, 2026. doi:10.1001/jamanetworkopen.2026.7997

Data
Additional Information: clinicaltrials.gov #NCT04585269
https://clinicaltrials.gov/study/NCT04585269
Data available: Yes
Data types: Deidentified participant data, Data dictionary
How to access data: The data that support the findings of this study are available from the corresponding author upon reasonable request, katie.devine@rutgers.edu.
When available: With publication

Supporting Documents
Document types: None

Additional Information
Who can access the data: researchers whose proposed use of the data has been approved
Types of analyses: for any purpose
Mechanisms of data availability: with a signed data access agreement
